# “There are many fevers”: Communities’ perception and management of Febrile illness and its relationship with human animal interactions in South-Western Uganda

**DOI:** 10.1371/journal.pntd.0010125

**Published:** 2022-02-22

**Authors:** Michael Wandanje Mahero, Katherine M. Pelican, Jacinta M. Waila, Shamilah Namusisi, Innocent B. Rwego, Charles Kajura, Christopher Nyatuna, David R. Boulware, Joel Hartter, Lawrence Mugisha, Cheryl Robertson, Dominic A. Travis

**Affiliations:** 1 Department of Veterinary Population Medicine, University of Minnesota, St. Paul, Minnesota United States of America; 2 Makerere University, School of Public Health, Kampala, Uganda; 3 College of Veterinary Medicine, Animal Resources and Biosecurity, Makerere University, Kampala, Uganda; 4 Hoima District Local Government Uganda, Hoima, Uganda; 5 Dept. of Medicine, University of Minnesota, Minneapolis, Minnesota, United States of America; 6 Environmental Studies Program, University of Colorado, Boulder, Colorado, United States of America; 7 EcoHealth Research Group, Conservation & Ecosystem Health Alliance (CEHA), Kampala, Uganda; 8 School of Nursing, University of Minnesota, Minneapolis, Minnesota, USA United States of America; Universite de Montreal, CANADA

## Abstract

Diagnosing the causative agent of febrile illness in resource-limited countries is a challenge in part due to lack of adequate diagnostic infrastructure to confirm cause of infection. Most febrile illnesses (>60%) are non-malarial, with a significant proportion being zoonotic and likely from animal origins. To better characterize the pathways for zoonotic disease transmission and control in vulnerable communities, adequate information on the communities’ experiences and lexicon describing fever, and their understanding and perceptions of risk pathways is required. We undertook an ethnographic study to understand behaviors, exposures, and attitudes toward fever at the community level. Our hope is to better elucidate areas of priority surveillance and diagnostic investment. A focused ethnography consisting of participant observation, informal conversations, 4 *barazas (*community meetings), and formal ethnographic interviews (13 Focus group discussions and 17 Key informant interviews) was conducted between April and November 2015 in Kasese and Hoima Districts in Uganda. Perception of illness and associated risk factors was heavily influenced by the predominant livelihood activity of the community. The term “fever” referred to multiple temperature elevating disease processes, recognized as distinct pathological occurrences. However, malaria was the illness often cited, treated, or diagnosed both at the health facilities and through self-diagnosis and treatment. As expected, fever is as an important health challenge affecting all ages. Recognition of malarial fever was consistent with a biomedical model of disease while non-malarial fevers were interpreted mainly through ethno etiological models of explanation. These models are currently being used to inform education and prevention strategies and treatment regimens toward the goal of improving patients’ outcomes and confidence in the health system. Development of treatment algorithms that consider social, cultural, and economic contexts, especially where human-animal interaction is prevalent, should factor animal exposure and zoonotic illnesses as important differentials.

## Author summary

Across the tropics malaria prevalence has been steadily declining over the last 15 years. This has revealed the importance of non-malarial febrile illnesses and their link to livestock production. Unlike malaria, managing NMFI in resource limited settings is challenging due to the limited number of diagnostic tools available and communities’ health seeking behavior. To develop effective NMFI prevention and management strategies, a comprehensive exploration of the socio-cultural drivers of NMFIs among rural livestock keeping communities is required. Towards this end we conducted a focused ethnographic study in Western Uganda among communities with high human livestock interaction to describe communities’ a) perception of the syndrome of fever b), important biosocial pathways associated with febrile illness and c) their management of these febrile illnesses. This paper describes important biosocial drivers of NMFI, highlights important aspects of the cultural epidemiology of NMFIs, and illustrates the unintended consequences of global health policies on the management of NMFIs at the community level. It concludes by proposing important additions to NMFI clinical algorithms that could improve the sensitivity of such tools in areas of limited diagnostic capacities.

## Introduction

The term “fever” has various definitions in different parts of the world. Strictly defined, “fever” is an elevation in body temperature that exceeds the normal average daily temperature (≥38.3°C/101°F) [1]. It is the primary observable characteristic of several diseases of global importance such as malaria, neglected zoonotic infections, as well as many invasive bacterial and viral infections [2–5]. In most resource limited settings, “fever” is also the major symptom compelling people to seek health care services [4]. Unfortunately, proper diagnosis of febrile illness in many countries is difficult due to delayed recognition and reporting of illness, the need to test for multiple potential pathogens and lack of adequate diagnostic infrastructure [2,6,7].

In resource-limited settings, lack of diagnostic infrastructure necessitates the use of presumptive treatment especially in peripheral health facilities. Thus, most non-malarial febrile illnesses (NMFI) are misdiagnosed and treated as malaria despite the decline in malaria cases among febrile patients in Sub Saharan Africa. This leads to increased drug resistance and poor clinical outcomes [8,9]. Studies across Africa have shown greater than 50% reduction in malaria-associated fevers over the last few decades; current prevalence rates range from 1.6%-37.0% depending on the area and age group [8,10–12]. However, despite the frequent assumption that febrile patients have malaria, especially in remote, rural and poor settings, there are many infectious diseases which present with a fever, ranging from self-limiting viral infections such as common colds to more fatal infections like viral hemorrhagic fevers (VHFs) such as Marburg and the recent Ebola Virus Disease outbreak in the Albertine region [13]. In fact, there is ample evidence showing that more than 60% of febrile illnesses in Eastern Africa are non-malarial [7]. One of the major contributors to fever in Africa, once malaria is ruled out, is diseases of animal origin or zoonotic diseases [3,7,14]. The rise in these zoonotic diseases is driven by a host of factors including the increased interaction of humans and animals [15].

Although often neglected, zoonotic diseases have a substantial impact on human well-being globally. The 13 top ranked zoonoses are estimated to cause 2.2 million deaths with 2.4 billion cases of illness annually [16]. Of these, 12 of the 13 are febrile illnesses. These include leptospirosis, brucellosis, hepatitis E, leishmaniasis, Q fever, rabies, toxoplasmosis, trypanosomiasis, tuberculosis, and food borne infections [3]. Unfortunately, despite their substantial contribution to morbidity and mortality, fevers related to these infections are likely to be misdiagnosed and mismanaged in the absence of adequate diagnostic facilities. Indeed, controlling zoonotic infections which circulate between animals, people and the environment presents conceptual and institutional challenges such as the need for effective multi-sectoral collaboration, paucity of tools for disease prioritization, diagnosis, inadequate surveillance and response systems, limited funding, and weak policy frameworks [17–20].

In Uganda the causes of NMFI are numerous and not well defined. A significant portion of these NMFIs are likely zoonotic in nature due to; the high human-animal interaction, rich biodiversity, and increasing human population. Uganda is prone to zoonotic disease outbreaks and vulnerable to the chronic impacts of endemic zoonotic diseases [21]. Close to 58% of Uganda’s population is engaged in livestock farming, with a significant portion of its rural population living adjacent to wildlife refugia and dependent upon natural resources for their livelihoods [21,22], further increasing the risk of zoonotic disease transmission through this wildlife-livestock pathway. Rural communities within the Albertine Rift in western Uganda clearly illustrate these challenges. This region is a mosaic of agriculture, settlements, natural resource extraction sites, national parks, wildlife and forest reserves and forest fragments [23–25]. This confluence of high biodiversity, high human animal interaction, rapid land use changes, pre- and post-colonial government land policies, fragile public health systems and unique socio-cultural practices provide an ideal environment for disease spillover events to occur [26–28]. These events have recently resulted in a number of infectious disease outbreaks within this region including Anthrax, Brucellosis, Ebola, and Marburg (VHFs), all of which could present as a mild to acute febrile illness with specific or non-specific symptoms [15,26,29].

Although global guidelines exist for managing the syndrome of “fever” in resource limited settings, there is minimal guidance on how to integrate location specific risk factors or local knowledge into diagnostic or treatment algorithms. For example, the current WHO Integrated Management of Adolescent and Adult Illness (IMAI) algorithms are recommended for adaptation at health centers with context (countries/communities) specific information regarding the causes of fever [1]. However, these adaptations are focused on testing and treating with little mention of the cultural context from which these diseases arise [17]. Our previous collective research has highlighted the need for greater understanding/ exposure of medical staff in these frontier communities to the cause, symptoms and management of zoonotic febrile illness (especially given the limited diagnostic infrastructure at these peripheral health centers), while at the same time describing the experiential knowledge and unique ecological patterns associated with these zoonotic febrile infections among these rural communities [22,30–33].

Therefore, in areas of high human-livestock-wildlife interaction, it is important to integrate questions that address this cultural epidemiology of NMFI into the clinical algorithms for the management of fever [34]. Previous qualitative studies addressing fever have had a narrower focus on malaria and did not highlight the unique nature of communities living in such human-animal interfaces [35], and their perceived most important risk pathways [27,36,37]. Given these complexities and the paucity of knowledge surrounding them, this exploratory study aimed to describe the cultural perceptions of fever and associated health practices among communities living in or around wildlife protected areas. We hypothesized that different socio-cultural communities recognize different forms of fever and used a focused ethnographic approach to describe the communities’ a) perception of the syndrome of fever, b) important biosocial pathways associated with febrile illness and c) their management of these febrile illnesses. The goal of our research was to understand local knowledge and observations about fevers. Our methods included deep engagement with a frontier population using ethnographic methods. This understanding will help health practitioners supplement their diagnostic efforts and inform prioritization of surveillance and diagnostic investments in areas of limited resources.

### Theoretical framework: Relational theory of meaning

We attempt to tap into and interpret communities’ intuitive and experiential knowledge regarding infectious diseases using the relational theory of meaning. The relational theory of meaning is informed by structuralism and posits that cultural meaning is created by symbols. These symbols derive their meaning from the manner in which they are used and related to other symbols in that culture [38]. Symbols within a culture include words, objects, places, or gestures/actions. Language is a primary symbol that encodes cultural meaning in every society and thus can be used to access or decode all aspects of a cultural group [39]. We used this approach to frame communities’ intuitive knowledge and their ability to interpret observations in their environments and predict future happenings.

Thus, we focused on describing folk terms (words) used by the participants and the main domains describing the symbols of importance (and their relationships) around the issue of health, with special emphasis on febrile illness [40]. The smallest unit of cultural meaning is the symbol, and these are categorized within broader ‘cover’ categories called domains. These symbols/individual folk terms contained within domains are referred to as included terms (for example, fruit is the cover term and apples, oranges and bananas are included terms). Using such a systematic discovery of meaning can reveal important elements of cultural knowledge [41]. Similar to Hoffman’s domain and taxonomic analyses of “response strategies engaged by women as they negotiate mothering across cultures”, and Spradley’s investigation of “ways to make a jug”, we provide an analysis of “kinds of febrile illnesses” in communities that have significant human-animal interaction [39,42]. We used this approach to identify episodes from the transcripts of interviews and observations that refer to different aspects of the experience, evidence, pathway, and management of febrile illness. The words and phrases used were recovered, analyzed and reconstructed into emergent patterns or domains as previously described [41,43]. It therefore allowed us to systematically unveil local definitions and understanding of fever as used within this cultural context. This emic description of febrile illness is situated within a syndemic model of health which recognizes the intricate deleterious connection co-presenting illnesses (such as malarial and NMFI) have with social and environmental factors where they are found [44]. Interpretation of the emerging analysis using inductive coding techniques was used to describe how these communities’ perceptions of febrile illness influence health behavior and thus ultimately the management of febrile illnesses [45].

## Methods

### Ethics statement

Ethical approvals were obtained from the University of Minnesota Institutional Review Board (IRB) study number 1502S64201, the Joint Clinical Research Centre (JCRC) IRB in Uganda and the Uganda National Council of Science and Technology (UNCST HS 1726). All tools and approaches used were reviewed and approved by UMN and JCRC IRB boards. Informed consent was obtained from all key informants and focus group participants.

### Study area

The study was conducted in two districts (Kasese and Hoima) in western Uganda which are located within the Albertine Rift Valley, an approximately 500-kilometer-long geo-formation that is part of the northern sector of the Western Rift. It stretches from the Kivu volcanic province in the south (1°S) to the border between Uganda and South Sudan in the north (3°N), straddled between latitudes 29°E and 31°E with an estimated area of 14,098.9 km^2^ [46,47]. The districts are characterized by both pastoral and agro-pastoral livelihood systems. The Albertine Rift Valley has rich biodiversity, high livestock density, a tropical climate with highly fragmented forest areas and rapidly growing urban centers.

Kasese has a population rate of over 6.4% and Hoima has 4.3%, making both among the fast growing and highly populated districts (Hoima population 572,986; Kasese population 702,029), in Uganda [48,49]. Kasese’s major pastoralist groups are the Basongora and Bahima who own majority of the 110,000 cattle in the district. While in Hoima the Bahima own most of the district’s 140,000 cattle. Both districts also have large agro-pastoralist populations which include the Bakhonzo, Batooro in Kasese and, Bakiga, Banyoro and Lugbara in Hoima.

The pastoralists inhabit the low-lying regions of Hoima and Kasese and are characterized by high reliance on livestock for economic and social wellbeing. These pastoralist areas are often semi-humid to semi-arid regions (annual rainfall ≤1000mm) and thus pastoralists employ various types of targeted mobility to access adequate pasture and water for their livestock. The vegetation cover is made of bushy-acacia trees, shrubs, and sparse grassland. Majority of cattle kept are *Ankole* cattle, a hybrid between *Zebu* (*Bos-taurus indicus*) and long-horn cattle (*Bos-taurus taurus*). Apart from *Ankole* cattle, few *Boran* and *Zebu* cattle are also kept with different herds mixing freely, and occasionally interacting with wild ruminants [50]. Families live in closely knit communities where all family members have roles and their interaction within this larger network of community members is governed by set of cultural rules and expectations that are adhered to closely. Most of the areas they inhabit are often remote, have limited infrastructure and border large water bodies or wildlife reserves. A lot of their former communal grazing land has been transformed into wildlife protection areas by government policies. This has led to increased human wildlife interaction and conflict, and a sense of marginalization [51]. Housing is often temporary to semi-permanent in nature (S3 Fig), the area sparsely populated apart from centroids of habitation next to water bodies or trading centers and access to healthcare or education is limited. In addition to livestock production, some pastoralists engage in limited auxiliary activities such as crop production or firewood collection to help complement their pastoral proceeds [51,52].

The agro-pastoralists live in areas that are higher in altitude; receive more rainfall (>1000mm) and have lower daily mean temperatures (18°C—30°C). The vegetation is savannah grassland interspersed with bushy shrubs and sometimes fragmented forests. The area is densely populated with better access to schools and health care centers (compared to pastoralist regions), most farmers practice mixed crop-livestock farming for both subsistence and commercial purposes. Increasingly, these farmers are adopting intensive production methods such as zero-grazing (stall-feeding system) and paddocking systems, while improving their indigenous breeds by crossing them with high yielding dairy breeds such as the Friesian cows. Land ownership is individual, and houses are mostly permanent [53,54].

The lakeshores of both districts are home to fishing communities (e.g., the *Langi*, *Alur*, *Baganda*) and, in some cases, pastoralist communities as well *(Banyankole/Bahima*, *Basongora*). The homesteads are often lined up in neat rows adjacent to the lake shore. Most have mud walls and grass thatched roofs with a few brick-walled and tin roofed houses. Amenities are often limited, temperatures high and access to healthcare poor. There is a lot of small scale trading because of the fishing industry, high rate of human-animal interaction because all converge around their need for water, high rate of in and out-migration, significant economic and social exchange between transient occupants of these communities, and as a result higher prevalence of HIV compared to other parts of the country [55]. The soils are sandy and hence cannot sustain crop agriculture well. These sandy soils make construction of toilets (pit latrines) difficult resulting in frequent waterborne disease outbreaks. There are many boats, people often use motorcycles to commute in and out of the village and a few vehicles (most probably belonging to local traders) can also be spotted picking or dropping merchandise. These communities are engaged in limited crop and livestock production for subsistence and small-scale commercial purposes.

In addition to agricultural production, Kasese and Hoima also have protected and non-protected forests with a substantial wildlife population. Hoima is also part of a biodiversity hotspot–the Albertine Rift–having one of the largest portions of unprotected forests in Uganda with high rates of deforestation averaging about 2.27% annually, three times higher compared to protected environments [48,56,57].

### Health system

To appreciate the current challenges faced by the peripheral public health system in these areas, particularly in the diagnosis and management of febrile illness (curative services), it is important to look briefly into the past and understand why this current need exists. Uganda’s health system has undergone tremendous changes over the last 60 years, driven by colonial policies, post-independence political turmoil, and subsequently, health sector reforms. During the 1960’s and 1970’s Uganda had a remarkable health system and one of the best network of health services in the continent, allowing its citizens to enjoy free access to health care services [58]. The political instability of the late 1970’s and 1980’s took a toll on the social, economic and health systems within the country, resulting in physical deterioration of health facilities and massive exodus of trained personnel. During its recovery period in the 80’s and influenced by global reform policies, Uganda invested in a primary health care community based approach, focused on maternal and child health and implemented through vertical programs that had little integration into the health system’s governance structure [59]. This was subsequently followed by the fee-for-service model for all curative services, and reduced government investment into the public health infrastructure particularly that which was located at the lower administrative level [58]. Despite subsequent efforts and polices to try and ameliorate the situation, these global and national polices of the 1980s and 1990s have resulted in persistent disparity in health access particularly among rural communities given the limited diagnostic and curative infrastructure in many of these peripheral public health units. The current health care delivery system mirrors the government administration system with health care delivery centers from the national to the village level, complemented by private-not-for profit facilities, private for profit health facilities and traditional medicine practitioners [59] (S1 Table).

In these frontier communities, most individuals seek treatment from a local health center II, or in some cases, health center III, and upon referral, the regional or district health centers if they have the resources required for this. The health center (HC) II is normally staffed by an enrolled nurse, a qualified midwife, and at least two nursing assistants. It is often built and staffed to handle out-patients with common illnesses and support maternal and childcare. The HC III has a bigger pool of professionals and is often led by a senior clinical officer and has a maternity ward and functional laboratory (S1 Table) [60]. Sometimes community members resort to local pharmaceutical shops or traditional healers for quick treatment alternatives as well. Kasese has 46 HCIIIs, 4-HCIVs, and four hospitals while Hoima has 33-HCIIIs, 3-HCIVs and one regional hospital. These health centers often have a shortage of drugs and qualified personnel to cater for the existing need on ground. Accessibility to these formal health services in Kasese is 78% and 94% in Hoima. Some of the barriers that result in this disparity include; geographical barriers, lack of drugs in public facilities, limited qualified staff and long wait times, as well as education and sociocultural barriers especially among the pastoralist community that is larger in Kasese [61]. The recorded leading cause of morbidity and mortality is malaria, followed by respiratory tract infections in both districts [62].

Most patient NMFI management at the peripheral health center level is syndromic given the limited diagnostic infrastructure at this level. Treatment is often guided by WHO IMAI for first level health facilities and focuses on malaria management. Greater diagnostic facilities are available at the district level where management is guided by the WHO IMAI District Clinician Manual. Yet even these resources could benefit from occupational and contextual (sociocultural) patient information to help improve clinicians’ diagnostic and treatment strategies [63].

### Study design

Ethnography is a discipline that seeks to describe a people’s way of life informed by a deep desire to understand their view points and interpretation of happenings in their environment (proverbial native’s perspective) [41]. It involves extensive data collection from multiple sources. The data sources used in our study were focus group discussions, key informant interviews, participant observations, archival data review (reports, district health information), community meetings and informal conversations. Our focus group meetings involved; i) a group of carefully selected individuals (preferably not known to each other hence selected from different households), ii) a moderator gently guiding the participant’s conversation down the questioning route (using questions and probes from the guide as needed) without dominating the conversation, thus allowing for group interaction that produced data and insights that would not have been accessible if it were not for this carefully set up context that was both permissive yet planned, iii) an observer whose work was to capture the salient non-verbal features of the interactions, and the community setting as well [64]. The in-depth interview targeted individuals who were knowledgeable about the communities’ way of life, health experiences and had some expertise in human, animal, or community health. Carefully selected questions were used to draw from their specialized knowledge and triangulate the FGD data. Participant observation was used to help contrast informants’ narratives with daily practices of the communities. It involved a close follow up on livelihood and cultural practices, human-animal interactions, level of health services available, available infrastructure and its influence on community well-being. By so doing it allowed us to make explicit, the rules, unspoken ideals, norms and values that are critical for the functioning of these communities [41].

Using these streams of data, this study employed a cross-sectional, qualitative, exploratory design and a focused ethnography (emic) approach to describe the syndrome of fever and important biosocial pathways associated with NMFIs. This method was appropriate given our desire to understand communities’ point of view, their vision and interpretation of happenings in their environment, social interactions and behavior around a narrow focus on health [39,65]. Selection of counties experiencing high malarial and high NMFI was guided by the district surveillance (archived) data. Additionally, communities that accounted for diverse livelihood strategies within the selected counties were purposively selected to explore unique community-based understanding of common febrile illness, their causes, their management, and their lived experience.

### District surveillance data

Given our desire to understand the spatial distribution of febrile illness occurrence in the two districts in order to guide selection of counties, surveillance data (archived) was obtained through the District Biostatistician’s office in Hoima and Kasese with permission from the District Health Officer of each district. This was health facility data routinely collected from public and private health facilities within the sub counties in each district and summarized using the District Health Information Software (DHIS2). The data collected included information on key indicators on communicable and non-communicable diseases. Data extraction was limited to communicable (febrile) diseases; Malaria, Tuberculosis, Typhoid fever, Severe Acute Respiratory Infection (SARI), Epidemic prone diseases (meningitis, dysentery, measles, Yellow Fever, VHFs, plague), Neglected tropical diseases (e.g., Schistosomiasis, Leishmaniasis, Lymphatic Filariasis, Onchocerciasis, Sleeping Sickness, Bacterial Zoonoses). All laboratory confirmed cases of Malarial and Non-malarial febrile illnesses among children above 5years old and adults during 2012 and 2013 were summarized and the average tabulated using MS Excel.

In Hoima district, 50% of rural sub-counties, especially those located near the lake, had high malaria prevalence, while 75% of those located in peri-urban areas and areas next to natural reserves had high prevalence of NMFI especially the neglected tropical diseases (NTDs). In Kasese district all the sub-counties recorded high levels of malaria and some typhoid infections (S1 and S2 Figs).

### Study participants and data collection

Data was collected over seven months in twelve sub-counties with evidence of moderate to high malarial and NMFI occurrence in Kasese and Hoima district (S1 and S2 Figs) that were identified using district surveillance data. In each district selection of these sub-counties was done to capture the diverse livelihood practices within each district. Data were collected using multiple methods, including participant observation, informal conversations, *baraza* (community meetings), focus group discussions (FGD) and key informant interviews (KII). This allowed for robust data collection and extensive cultural context from the researchers’ personal experience living and interacting with members of these communities.

The FGD and KIIs guides had open ended questions that were used more as discussion prompts and guides. Development of the guides was based off the One Health principle that posits human, animal, and environmental health are dependent and inextricably linked [66]. We also gleaned from some of the preliminary studies we had conducted in the area, and other relevant research regarding health defining human-animal interactions to help inform the design of the guides (S4 and S5 Texts). The guides were designed to start with a set of “grand tour” descriptive questions to get an idea about the cultural scene followed by “mini tour” questions to narrow down to more specific items, while probing for “native” terms and phrases [32,66,67]. All sessions were recorded to facilitate easier and more accurate transcription.

Although not from this community, the first author is of East African origin, has a working knowledge of the *Runyoro/Runyankole* language. He is a veterinarian who has lived in and has extensive knowledge of these agricultural communities. He therefore uses his knowledge of the region and these communities to facilitate better connection and dialogue with the communities without losing the in-depth appreciation of the cultural context and how it informs the emerging themes. Allowing him to effectively draw upon communities’ interpretation of their lived experience, and to examine how communities’ livelihoods and culture influence risk, perception, and management of febrile illness. As a result, his epistemological assumptions are consistent with that of constructionism.

Key informants included human, animal and environmental health professionals and administrative officials from Hoima and Kasese, selected based on their technical knowledge and cultural understanding of these communities individual informed consent was obtained before each KII was conducted (S7 Text). FGDs were conducted at a site normally used for village meetings away from other community activities or distractions and comprised of male and female community members from multiple social strata, cultural backgrounds, occupations, and adult age groups (Table 1).

**Table 1:** Sample Characteristics by sex, region, livelihood, and profession (*N = 206)*.

	Kasese^a^			Hoima^b^		
	Male (n)	Female (n)	F.G./K.I.I (n)	Male (n)	Female (n)	F.G./K.I.I (n)
**Focus Groups (**Livelihood)						
Pastoralism	25	5	2	9	3	2
Fishing/Salt Mining	14	6	1			
Agro-pastoralism	8	9	2	42	14	3
Fishing/ Agro-pastoralism	15	15	2	4	11	1
**FGD Totals**	**62**	**35**	**7**	**55**	**28**	**6**
**KII Interviews** (Profession)						
Health/Clinical Officer	2	2	4	3		3
Veterinary Officer/Production Officer	2		2	2		2
Local Council III/IV Administrator	2		2	3		3
Development Officer		1	1			
**KII Totals**	**6**	**3**	**9**	**8**	**0**	**8**

^a^Focus groups and IDI were conducted in 6 sub-counties in Kasese {Kyarumba, KatweKabatoro, Lake Katwe, Munkunyu, Kahendero(Muhokya), Kasese Central

^b^ Focus groups and IDI were conducted in 6 sub-counties in Hoima{Kigorobia, Kiziranfumbi, Bugambe, Bujumbura, Buhimba, Kahoora} Two Community Outreach Meetings (Barazas) were held in Hoima (Kiziranfumbi and Kigorobia)

Summary of Kasese participants in grey.

Prior to holding FGDs in any community, we first met with local council officials (these are the gate keepers in these communities) and a few community members. We would then proceed to explain the basis of the study and its potential benefits, inclusion criteria for participants and answer questions they had. After which we agreed on a suitable day for the focus group to be held. We then left them with translated explanations of the study and its objectives. This small group of opinion leaders reached out to other community members using a snowball approach and extended our invitation without any form of coercion or undue pressure.

Participation was voluntary and recruitment was supported and guided by these local council chairpersons from selected communities. Only willing individuals who were 18 years or older were invited to the focus group. Before each session verbal consent from the group was sought (S8 Text). This included an explanation of the study objectives, any risks/benefits, and a commitment to ensuring confidentiality of all that was discussed. During the discussion no names or personal identifiers were used. Only the moderator was allowed to tape the proceedings and recordings were downloaded unto a secure laptop that was encrypted for extra security. Participants were allowed to excuse themselves from the group before or at any point during the interview if they did not consent to participation. FGDs lasted about 60–90 minutes and were led by one of the team members trained in ethnographic methods and fluent in the local language.

Selection of livelihood groups (pastoralist, agro-pastoralists, fishing) was based on their distribution and availability in the two districts. Balance between male and female participants was desired but dependent on the community. Combined sessions were deemed wise by the community leaders to reduce any suspicion among some of the communities targeted. Therefore, interview teams paid particular attention to create space to hear from the women during the interviews. This was particularly necessary in the pastoralist community. Among the agro- pastoralists and some of the fishing villages the women were more vocal than the men. In this case effort was made to get balanced perspectives from the men as well. Data saturation was assessed through evaluation of the FGDs and KIIs for increased repetition of emerging ideas across the groups.

Participant observation was conducted during formal interviews and informal interactions with community members to help make more general observations about the communities and glean additional information on the health behaviors and human animal interactions. Observations were made in several settings: 1) fishing villages and fish landing sites, 2) pastoralists’ grazing and cattle watering areas 3) within the homesteads of study participants 4) trading centers 5) health centers 6) at village meetings. First author, SN, CN, CK, LM, and other study staff conducted the observations. A total of 400 hours of observation were completed from January 2015 to August 2015 (SI: Field notes). First author undertook the role of a veterinarian and adopted an observer stance as participant-as-observer because it facilitated an in-depth understanding of the various human livestock interactions and its possible influence on health.

Interpreters were research team members who were; fluent in the local languages of *Runyoro*, *Rutooro* and *Runyankore* (these three languages are similar and are all part of a larger cultural group called *Runyankitara*), trained in ethnographic methods and research ethics involving human subjects. The research team involved in conducting interviews had 2 veterinary technicians, 3 veterinarians, 1 clinical officer, 3 social scientists all with experience in community health and social/behavioral research. The inclusion criteria included a willingness to participate, residence in the study area for more than a year and a minimum age of 18 years and from different households within the village.

Permission to access the community was also obtained from the districts’ chief administration officer, the sub-county administrators, and local council leaders at the community level. Given their gatekeeper role these leaders at the community level guided our approach into the community. In some communities the trusted gatekeepers were the local nurses and village health team leaders, and thus they were also consulted and coopted into our community entry teams. Often entry into the community was initiated by having a local village leaders meeting (*baraza*) where the goals and value of the research were clearly laid out and their collaboration requested. Preliminary community concerns around the issues of health and livestock productivity were also addressed as they emerged. This helped the research team get a sense of the felt human and animal health concerns within these communities.

### Data analysis

All recordings were independently and directly translated from *Runyakitara* to English and transcripts from the FGDs and the KIIs compared to the recordings for consistency and accuracy of translation. Initial data analysis and summary was done using an inductive content analysis approach [64]. To achieve this, a coding framework (S2 Text) was developed by MM with CR guidance and input by reading through the transcripts to get a sense of the whole and identifying the ideas (codes) consistently emerging through the transcripts using an inductive in-vivo approach. This first stage of open coding was both data driven and theory (One Health) based [65]. MM and JW reviewed the framework and made improvements to ensure consistency and integrity of the data collected. The analysis involved coding, categorizing of related codes and identification of themes. Regular check-ins with some of the team members involved in the interview process was done to ensure that coding and resulting patterns reflected the experience of the interviewees. The entire transcript was reviewed, and categories relevant to the research question described.

Further ethnographic analysis was guided by our research objectives and the “relational theory of meaning” [38]. Spradley’s levels of analysis, domain and taxonomic analysis, were used to identify patterns in the data that make explicit, implicit cultural meanings of febrile illness and their relationships as previously described. This was achieved first by organizing the copious amount of data into categories (domains) and identifying terms that describe these categories (included terms). We identified the domain focus as the different aspects of febrile illness as experienced by community members (the nature, pathways/causes, and management of fever). After which the semantic relationships between these categories were assessed using the domain analysis framework [40]. The following universal semantic relationships; strict inclusion, cause-effect, attribution, were used to unpack the different aspects of the term *omuswijja* as used by community members [40] (Table 2). Field notes from participant observation were used to contextualize and guide interpretation of our findings (S3 Text). We used different sources of data (FGD, KII, and PO) to complement and clarify the emerging themes and patterns of data. To ensure rigor, regular briefing with CR, JW, KP, LM, DT and SN was done to identify and address potential biases. Additionally, regular reflection on the transcripts and audiotape recordings to ensure all emerging themes were captured and use of analytic memos during the coding process was also done. We used Atlas.ti (Atlas.ti Scientific Software Development Product GmbH version 7.0.82.0), a qualitative analysis software for data management.

**Table 2:** Summary of Spradley’s Domain and Taxonomic Analysis of FGD and KIIs from Hoima and Kasese.

Domain Cover Terms	Semantic relationship	Included Terms		
**Nature of febrile Illness** (*Omuswijja*)	**Is a type of fever** (Inclusion)	**Seasonal Fever** Mango fever, Maize fever, Mosquito fever, dry season/*obweire wo musana* fever, wet season/*obweire we njura* fever.	**Environmental/Poverty related Fever** (*oburofa*) Related to one’s environment and poor hygiene (*oburofa*) Fever related to exposure to the sun for long periods.	**Milk Fever/Cattle Fever** (*Omuswijja gwe nte*)
**Course of febrile Illness** (*Omuswijja*)	**Characteristics of fever** (Attribution)	**Symptoms** *“Our stomach swells because we drink the water and even we experience a general body weakness, headache (omutwe)*, *fever (omuswijja)*.* *.* *.*”*	**Severity** Mild illnesses that is self- limiting, responds to herbs or self-medication *“Severe febrile illness results in*, *CNS involvement e*.*g*., *stiff neck or convulsions in children*. *Requires one to go to the referral hospital and could lead to death”* *“Most of the fishermen cry chest pain and others cough blood the stomach swells”*	**Frequency/duration** Acute fevers- *(Omuswijja gwa amanyi) that* respond to treatment, Chronic fevers *(Omuswijja ogutakira)* that are often not diagnosed and thus never resolve *“Fevers are there all the time”*
**Folk recognition of febrile illness** (*Omuswijja*)	**Pathways of Febrile illnesses causation** (Cause-effects)	**Water pathway** Related to poor hygiene-lake used as latrine; wild animals spread disease through this… *“I am a “mubalia” or fisherman*. *When I go in the lake*, *I drink that same water which I use to ease myself…”*	**Livestock or animal pathway** *“Yes*, *yes very much because these animals sometimes drink from the same sources with us so they can give us flu or other diseases”*	**Vector Pathway** *"There is a fever that comes when the animals come back from the bush (park/forest) carrying flies"*
**Management of Febrile illnesses** *(Omuswijja)*	**Types of Management** (Inclusion)	**Visit Hospital** *“When we are sick*, *we go to the health center”* *“Availability of medicine in our health center is also inconsistent because most of the times we find no drugs at the health center”*	**Herbal medicine/Prayers** *“Sometimes we use omubirizi a natural herb and it also cures malaria”* *“We feel headache*, *fever and we use natural herbs first then when things fail*, *we go to the health center to get tablets but sometimes we do not have time to go to the health center”*	**Zoo medicine** (Use of animal by-products as medicine) *“Yes*, *that urine helps in curing cough and fever…ha ha ha yes we use fresh warm cow urine to control cough*.*”*

## Results

Our results are presented in a conical format starting with the grand tour of the health experiences of our informants, funneling deeper into the question of febrile illness, its perceived associated pathways and management strategies. Therefore, this section is organized into two main sections; i) Perception of illness, ii) Taxonomy of febrile illness (types/domains of; fever, pathways of fever and management of fever). A total of 206 individuals were interviewed from the two districts all aged between 20 and 65 years of age. Most of these were part of the focus group discussions (Table 1).

### Perception of illness

Illnesses and associated risk factors were perceived as having a medical or ethno etiological cause. This perception was influenced by the predominant culture or livelihood activity of the community. These illnesses were sometimes linked to specific etiological processes like viral or bacterial infections such as *Anthrax*, or more abstract metaphysical elements such as the influence of a Supreme Being, human agents of the supernatural or nature spirits such as malignant spirits of the wild, arising from a failure to appease these forces of nature during hunting expeditions as depicted by the observations shared by some of the informants (S2 Table). For example one participant stated, *“In some areas people believe that they have been bewitched in times of outbreaks e*.*g*. *in X community during the cholera outbreak”* (KII -senior health official-pastoralist community). While others shared that:

*In the past such things were there*, *I also used to witness people tying a banana fiber around the stick or spear so that the curses of the dead animal do not cause febrile illness*. *However today people have received salvation*, *so people just pray and get healed*. (FGD male participant agro-pastoralist/ hunting community).

*We use local herbs*, *but prayer is always the first*. *In most cases we use the venoniya tree (ekibirizi) for malaria*. *We squeeze it and get that juice*. (FDG female participant–agro-pastoralist community).

*Anthrax has also finished our animals we think it is transmitted by the wild animals because they share grass in the grazing field*. (FGD male participant -pastoralist community).

Participants from the five pastoralist FGDs (Table 1) were aware of animal related pathways of disease exposure and transmission, while the fishermen perceived lakes as both a source of blessing, in the form of the fish they caught, and a curse, for being a source of waterborne diseases. In the agro-pastoralist communities, illnesses were associated with changing seasons, different agro-ecosystems and exposure to vectors while working in their gardens.

The term “fever” referred to multiple disease processes which were often recognized as distinct pathological occurrences with unique presentations manifested by perceived elevated body temperature. These different illnesses could potentially be misdiagnosed by healthcare-workers given the blanket emic description, *Omuswijja*, used to describe all these conditions- (S2 Table). One focus group participant from the pastoralist community described this by stating… *“There are many fevers*” while another was quick to clarify that all febrile causing illnesses are called “o*muswijja”* (fevers). In general, the pastoralist communities referred to fevers that never heal, often linked to their interaction with cattle and were present all the time. They also described certain fevers that result in coughs, are seasonal and mirror similar signs in their cattle and goats (S2 Table), as revealed by this participant’s observations:

*Yes*, *we also get fever and flu signs (Ekihinzi) during certain periods of the year …everyone seems to get it and at the same time we also see these signs in our cows and especially the goats*. (FGD female participant-pastoralist community).

However, malaria seemed to be the febrile illness often cited, diagnosed, or treated, and was distinguished sometimes by referring to it as the mosquito fever- “*Omuswijja gwe emibu”*, or described using the borrowed term malaria- “*Omuswijja gwa* malaria”, however it was often simply called *“omuswijja”*. Community members were familiar with malaria symptoms and commonly available drugs used for treating malaria such as Coartem (Artmether/Lumefantrine) and Panadol (Paracetamol). An agro-pastoralist focus group participant’s sentiments reveal this reality *"Malaria fever is the most common illness in this area”*. While another affirmed that they do get help when faced with malarial fevers:

*We have a health center II it’s where we go and we are given drugs to swallow and we get better*, *we are usually given Coartem and Panadol [only]…but it helps us*- (FGD female participant agro-pastoralist).

As a result, malaria treatment was the first line of management for all febrile illnesses. Community members were aware of these limitations, and some were worried that most febrile cases were misdiagnosed and mismanaged. As revealed by sentiments from one of the participants from the fishing villages:

*Even if you have any disease only malaria is mentioned…you find us talking of malaria*, *yet we could be suffering from other diseases*. (FGD male participant-pastoralist, fishing community).

Concerns that were shared by participants from the agro-pastoralist community as well:

*The services (at the health facilities) are not good*, *there is only Coartem and Panadol* (FGD male participant, agro-pastoralist community).

Therefore, the local term *omuswijja*, although often interpreted as and in some cases used in reference to, malaria does not primarily refer to infection with plasmodium parasites. This complexity in the use of the term is well described by a participant. *“We suffer from fever [omuswijja] because some of us do not boil water or milk that we take and that’s why our health is usually not good*.*”* (FGD participant-Pastoralist community), possibly alluding to enteric infections such as typhoid fever or milk borne illnesses such as brucellosis.

### Taxonomy of Febrile illness

The domain and taxonomic analysis of febrile illness allowed for a transition from the grand tour description of illness in this community to a thick description of the communities’ lived experience with febrile illnesses. Four major domains emerged and will be highlighted in this section; i) nature of fever, ii) course/attributes of fever, iii) pathways of fever causation, iv) management of fever.

#### Domain 1: Nature of fever

Most participants drew strong cultural, livelihood, environmental or seasonal associations with the occurrence of fever, and used this knowledge to describe several categories or syndromes of fever (Table 2). These fevers differed in etiology, symptomatology, and duration (acute-chronic). In this section we unveil the different categories of fever as understood and explained by the informants and participants.

*Seasonal fevers*: Participants described a bimodal pattern to the occurrence of fever, which emerged during the rainy season linked to the increase in vectors, overgrown bushes, and stagnant water. Fever was also seen during the dry seasons. Sometimes presenting with respiratory symptoms attributed to a dusty environment or gastrointestinal symptoms due to a shift in their diet from milk based to a fruit/vegetable-based diet. Additionally, participants repeatedly noted that malaria or severe malaria occurred during the maize and mango seasons:

*During maize season people get severe malaria…during rainy seasons we experience malaria cases due to breeding of mosquitoes*. (FGD female participant agro-pastoralist community).

Some participants described fevers that seemed to persist throughout the year, affecting all age groups:

We *also have fevers all the time*. *Still connected to the use of water that is not hygienic…and presence of many flies in Runga [village]…Runga is dirty…* (FGD participant-pastoralist/fishing community).

*Environmental/Poverty related fever*: Poverty was commonly described as a reason behind the propagation of fever. Poor sanitation due to inappropriate human waste disposal and inadequate public health education was associated with the stomach fever, “*omuswijja gw’omubyenda”*. Some decried the limited presence of community health workers who were often relied upon to advise and encourage proper hygiene and sanitation in the community. Community members also seemed to depend on these community workers for guidance on identifying the causes, risk factors and prevention strategies of these febrile illnesses:

*The major cause of fever is poverty*. *How*? *For example*, *bush*, *a poor person cannot afford slashing*, *cannot afford putting up a good house…Other causes include stagnant water*. *Here sanitation is still poor*. *We do not see health personnel*. (FGD male participant–pastoralist).

Poverty was also seen as a barrier to constructing adequate housing and maintaining a clean environment devoid of unwanted bush and thicket. Participants identified this as a way to prevent exposure to vectors and parasites that would result in febrile illness. Some of the pastoralists felt marginalized because of their cultural and socioeconomic status, resulting in discrimination at some of the health care facilities they access, and a perceived poor management of their febrile illnesses and hence persistent fevers as one participant reported:

*Sometimes we are discriminated at the health centers*, *special attention is given to the people they know*. (FGD male participant-pastoralist).

*Milk Fever/Cattle fever*: Participants related fevers to food, particularly animal related foods. It seems that there has been an increase of these fevers over time, a phenomenon that is still not well understood especially among the pastoralists. Some believe this increase is due to increased movement of people and interaction of their livestock, because of dwindling pasture and water resources for extensive cattle rearing. Participants further identified consumption of raw milk as a cause of fever. This fever was related to an illness that also causes abortion in cattle, and often referred to as the *cattle fever “*Omuswijja *gw’ente”*. Participants verbalized that this was a new occurrence (sentiments that were independently verified by some of the health personnel during the key informant interviews). They indicated that, their fathers and grandfathers consumed raw milk without acquiring these diseases, but all seems to have changed,

*…Now when one takes milk without boiling the chances of getting a fever are high*. *Cough is also killing us especially the men who stay with cows all the time we don’t know whether we get it from cows or milk*. (FGD participant-pastoralist community).

These sentiments were also shared by some of the health professionals in these regions as depicted by these sentiments from a healthcare worker from an agro-pastoralist community,

*Yeah*, *they have febrile diseases such as TB because some of their cows are usually sick so they get TB from these animals…When people move they move with their cattle and along with them-diseases… We have seen an uptick of brucellosis (milk fever) in the district because of this*… (KII District health officer-agro-pastoralist community).

#### Domain 2: Course of Febrile illness

*Attributes of Fever (Symptoms and Severity)*: In most communities, there were symptoms attributed to the syndrome of fever (Table 2). Participants often conveyed a gradient of fevers depending on the severity of the illnesses yet did not always make this distinction in the use of the term”o*muswijja”*. Some were mundane illnesses that hardly prevented one from their daily routine; others were acute in nature associated with symptoms such as elevated temperatures, headaches, malaise, and body rigors. While others were associated with severe symptoms including painful joints, stiff neck, stomach pains, jaundice, and a prolonged course of illness, a few were linked to convulsions especially among children. One participant explained,

*The body becomes hot*, *changes in moods*, *physical pain*, *and the eyes become yellow*, *and one vomits yellow things*. (FGD male participant-agro-pastoralist community).

Other fever attributes discussed by participants included the frequency of febrile illness and factors driving this variation in frequency and duration as highlighted in Table 2.

#### Domain 3: Pathways of fever

Participants attempted to interpret the variations in fevers and ascribe etiologies to the different symptoms by linking them to different causative pathways (Table 2). We report these pathways in the order of emphasis laid by the communities.

*Water pathway*: Given the lack of potable water in most of these communities, many rely on surface sources of water. These sources of water hold strong cultural meaning, shaped by the need for place dependence and identity as described by this participant from one of the fishing villages,

…*Me “J” and my great grandparents were fishermen and so fishing is our major source of income…*. *I rely solely on the lake to cater for my needs as a fisherman*, *and my family needs*. (FGD male participant-fishing village).

Unfortunately, complex human and animal interactions that result in degradation of such hallowed spaces, which include, waste disposal and congregation of different species in search of water for drinking and wallowing, facilitate inter-species disease transmission and creation of conditions suitable for disease vectors such as mosquitoes or snails (S4 and S5 Figs). Community members from an agro-pastoralist and fishing community capture this issue very well:

*We have water fever “Omuswijja gwa mazi” all the time… yes water fever is the most severe kind of fever*. (FGD participant-agro-pastoralist community).

*On top of that*, *animals like hippopotamus urinate in the water and yet some of these animals are affected by certain diseases and hence some people end up taking water which is already contaminated by the animals’ urine; as a result*, *we get diseases like yellow fever*, *malaria fever*, *and cough*. (FGD male participant, fishing village).

The consistent contact with snail infested water was also described as a source of febrile illness. In many cases these fevers were linked to schistosomiasis infection *“Bilharzia also usually comes”* (FGD male participant—fishing village).

*During the dry season when there is no water all animals converge from various locations*, *they converge at these water sources so there are usually wrangles for these water sources at the same time there is spread of diseases among the livestock*. *For example*, *during these dry seasons all animals converge at the lake since the lake is for everybody and so [zoonotic] disease transmission occurs here*. (KII-Senior district agricultural officer).

*Livestock or Wildlife pathway*: Community members reported frequent interaction between humans and livestock, including ‘normal’ husbandry activities often seen as harmless and beneficial such as grazing or milking. In some cases, more intimate interaction such as, sharing living spaces with goats, pigs and poultry was also reported (S3 Fig). This was to protect their livestock from wild animals and thieves. Some felt this intimate interaction was benign while others believed it posed a big health risk, especially when sharing living quarters with poultry. Furthermore, sick animals with poor prognosis or chronic conditions were sometimes slaughtered for food without inspection by public health personnel, motivated by the need for protein and fear of economic losses.

In general, domestic animals were viewed as beneficial, clean and disease free unless they interact with ‘disease’ carrying wildlife, which were said to transmit febrile diseases such as anthrax and trypanosomiasis, and were described as a nuisance, destructive, disease harboring, dangerous, parasite-infested, and ‘hateful’. Additionally, wild animal bites and scratches were also associated with febrile illness. With an increase in population, change in land use resulting in a reduction of unprotected forests, and expansion of extractive industries, participants repeatedly commented on the destruction of the wildlife habitat, and increased interaction between humans and wildlife as illustrated by an informant’s comments:

*People do encounter wildlife because of the encroachment and degradation of the environment*. *For example*, *we have a chimp that picked a child from the garden*… *If managed well we can improve the area and make it a tourist attraction…If the chimp is king and man is king then there shall be co-existence and mutual benefit*, *but if the chimp is king and man is not…then these conflicts will persist*. (KII-District Veterinary Officer agro-pastoralist community).

Interestingly, rodents in the home or peri-domestic environment were not considered wildlife or harmful, they were fondly described, hunted often and even considered “domestic” animals. Consumption of raw meat during hunting expeditions was also reported. Hunters would consume raw venison, particularly the liver, while it was hot and fresh in the forest before returning home.

*It is often said that the hunters eat the raw liver while it is hot and fresh in the forest during their hunting expeditions…* (KII—District Veterinary Officer).

Dogs were also seen as vectors of diseases (such as rabies) and parasites from wildlife to humans according to several FGD participants and key informants:

*Oh yes we do have dogs and they even fight with these wild animals sometimes…bringing back diseases*. (FGD Participant agro-pastoralist).

*Dogs are often a species that interact with these wild animals…as a result many hunters are prone to rabid animal interaction*. (KII-District agricultural officer),

Another key informant comments underscores the importance of this animal link as a source of febrile illnesses by highlighting some risk practices prevalent within the community:

*When a cow aborts*, *I have witnessed the pastoralists consume the fetus and they say it is delicious*. *Yet you may not know why the cow aborted and this spreads diseases*. *They are fond of carrying out delivery with their bare hands this may cause disease as well*. (KII-agro-pastoralist community).

*Vector pathway*: Participants associated fevers with exposure to vectors; often linked to environmental, livelihood and social factors as described by a participant.

*For example*, *the domestic animals like cows come with flies and small mosquitoes from the bush that bite us*, *and we get sick of fever*. (FGD participant—pastoralist community).

Crop growers also encounter disease transmitting vectors such as biting flies (*Black flies*/ “*Biramfunzi”*) in the course of their farm work, especially during the rainy season. Furthermore, failure to embrace vector prevention and control measures increases the risk of febrile illnesses among community members as well illustrated by a participant’s sentiments.

*Usually for malaria the government gave us mosquito nets however some people misuse them…they leave them on the side at night instead of using the net*… . *they would complain that it is hot*. *As a result*, *we always have fever among us*. (FGD participants-fishing/pastoralist community).

#### Domain 4: Management of fever

*Utilization of the Formal Healthcare System*: Although participants acknowledged using public healthcare facilities when faced with febrile illnesses, it was evident that the long distances one had to travel on poorly constructed roads (many would talk of a journey of 8–10 kilometers to their nearest health facility), combined with the lack of medication and long waiting times at the public health centers, encouraged reliance on private chemists and drug shops, often with little diagnostic information to guide this self-medication (S2–S4 Tables). About 90% of the participants indicated a reliance on non-biomedical interventions especially for managing NMFIs.

*Here we are living at the mercy of God*, *why am I saying this is*? *Because the health center is usually empty of drugs you go there sometimes*, *they tell you no drugs…no drugs and some people end up dying so God is our trusted medicine*. (FGD female participant—agro-pastoralist community).

Of concern is the fact that some of these perspectives were also shared by health workers as described by one of the key informants:

…*the problem is that some of the health workers also believe in some of these traditions as well*. *I remember hearing one health worker at the hospital whisper into the ears of parents whose child had cerebral malaria…*, *this one is serious you need to take your child to brother z for prayers…*, *yet the child was responding well and just needed more time at the hospital*, *I encouraged them to stay…*(KII-senior district administrative officer-agro-pastoralist community).

Community members further emphasized that their reliance on these non-biomedical treatment options is because of challenges of getting accurate diagnosis or timely treatment as illustrated by the sentiments below:

*Sometimes we get fever all the time*, *and when we go to the hospital for checkup*, *they do not find malaria or anything*, *so they say*, *go back home you are fine*, *yet you feel feverish all the time…* (FGD male participant—pastoralist community).

*They only give us Coartem and Panadol at the health center and sometimes it is not available*, *so we buy and if you don’t have money you stay with your malaria*. (FGD female participant—agro-pastoralist community).

These community member sentiments were corroborated by some of the key informants’ observations as illustrated by these excerpts,

*Here we have two health centers and they are located nearer to the community so this has helped people to access the health services but the only challenge we face is that most times people do not find the drugs and this makes them get a negative attitude so people’s behavior tends to be negative towards the public health system because they usually find no drugs at the health centers*. (Female Health-In-Charge of sub district-agro pastoralist community).

*Most febrile illnesses are caused by malaria*, *typhoid and livestock associated fevers*. *Greatest challenge with the issue of non-malarial illness is diagnostic*. *Not only is there a challenge in diagnostic capacity but also the long time taken to identify some of these diseases is of concern…You find someone goes to the health center III one week two weeks …no change… anytime the disease is not clear or progress or recovery is not experienced*, *they resort to witch doctors…They also go to herbalists if they fail from the health center IIIs…*.*for example victims of rabies…*.*when they cannot get help at health center III they resort to herbalists*, *brucellosis the same thing*. *In fact*, *I know of a herbalist who claims they are able to treat brucellosis…* (KII-Senior district agricultural officer).

Across all livelihood groups the hesitation to use the formal health systems was driven by individuals’ experience with the health system or their perception of febrile illness in addition to the issue of cost as summarized in Table 3.

**Table 3:** Febrile Illness Experience and Perception across different FGDs and its Influence on Health Behavior.

Sociocultural group	Perception of Febrile Illness or Health system ability to Manage Febrile Illness	Perceived Barriers and Benefits of action	Influence on health Behavior
Agro-pastoralists and fishing villages, pastoralists	Fevers are a big concern among children may lead to convulsions and in some cases death. Sometimes linked to malaria	Quick action required to help child survive. Village health teams provide quick first aid for children especially those affected by malaria	Seek quick care from VHT and if referred to, the nearest competent health center to get help for the child.
Pastoralists	Fevers are linked to contact with livestock	Health centers are far, it is costly to get to the health centers, even when able to reach find long waiting times and often no drugs to resolve the febrile illness apart from malaria drugs	Resort to zoo medicine such as the use of urine. Sometimes visit village health team member or private drugs shops of private clinics
Pastoralists	Fevers are many and hard to distinguish	Health centers do not have the diagnostic capacity to help distinguish fevers and so treat only for malaria	Resort to herbal or zoo- medicine and if all fails consult religious leaders for prayers. Sometimes seek care at referral hospitals
Agro-pastoralists	Most common fever is malaria (omuswijja gwe mibu), especially during the rainy season.	Herbal medicine is a viable option easier to get and cheaper e.g., *orutotoiyma*, (*Lamiaceae* spps) *aloevera*	Resort to herbal medicine first and then turn to formal health care systems if fails
Fishing villages	Fevers are sometimes linked to stigmatizing diseases like HIV	Stigma from being associated with sexual diseases	Refuse to disclose full extent of illness. Link their symptoms to malaria and seek help for malaria
ALL	Febrile illness are unavoidable	No vaccinations or preventive strategies to prevent febrile illnesses in adults	Only vaccinate children. Do not invest in prevention among adults
All	Febrile illness have multiple causes	Health centers unable to diagnose and even when they do most drugs are not available. Sometimes they require multiple trips for the management of different illnesses diagnosed	Resort to self-medication or seeking treatment from informal health care providers.
Pastoralists	Some fevers are chronic they never resolve	Health centers do not know how to deal with these fevers OR discriminate against us	Seek no care, self-medicate or resort to herbal medicine

*Ethno medicine*: The use of traditional medicine was common. Its nature and frequency was greatly influenced by the communities’ perception of illness, livelihood activities and cultural norms. Whereas the use of animal waste such as cow dung and urine among the cattle keepers emerged as a common phenomenon, crop farmers mostly used local herbs for febrile illnesses.

The detail and use of these animal by-products stemmed from years of community observation and the folk understanding of these febrile illnesses that seemed to impute a feeling of relief from the use of these products. Emphasis was placed on time and method of urine collection; cow urine was recognized as having therapeutic properties and consumed when still warm. It was mixed with cow dung or milk depending on the symptoms. Participants described the purgative, anti-pyretic, antimicrobial and immunomodulation effect of these ethno-zoological products with detail and conviction, often these products had several uses in addition to the treatment of fever.

*The animal wastes e*.*g*., *cow dung and urine are very helpful to us*. *For example*, *cattle urine cures fever “omuswijja”*. *We also mix up cow dung and take like a liter and this helps cure ‘malaria’*. (FGD participant—pastoralist community).

*Yes*, *for example*, *cattle urine is used in cleaning of stomachs especially in children*, *what we do*, *the urine is tapped from the cow when it is still warm*, *and it is given to a child there and then*. *Finally*, *the stomach is cleaned and in the end*, *it treats fever and new flu (Influenza)*. (FGD female participant—pastoralist community)

Community members also identified plants used to manage febrile illnesses in both humans and animals, and at times were given priority over biomedical interventions or augmented with prayer:

*Herbs differ we know there are two types*, *there are those which require bloodshed*, *we don’t use those but these ones where you get leaves or roots of a plant and mix with water*, *those ones have no problem*.(FGD participants agricultural community).

*We feel headache*, *fever and we use natural herbs first then when things fail*, *we go to the health center to get tablets but sometimes we do not have time to go to the health center*. (FGD male-participant pastoralist).

*Me I usually use natural herbs like “Omubirizi”* (herbal extract from the *Venoniya* species). *I take like two cups*, *and I am fine like in two days*, *so I don’t usually go to the health center for drugs*. (FGD male participant–agro-pastoralist community).

*We use local herbs*, *but prayer is always the first*. (FGD female participant—agro pastoralist community).

## Discussion

This study revealed that different sociocultural communities experience and recognize different forms of fever given the different livelihood activities and thus risks they are exposed to. We describe how these different rural communities along the human- livestock-wildlife interface in western Uganda negotiate meaning of their febrile illness experience through a cultural lens. Given the lack of “health infrastructure”, perceptions and attitudes toward the cause, severity and control of fevers are important aspects of the eco-ethno-epidemiology of disease in this setting. Some of these perceived causes of fever are consistent with the Epidemiology Triad of disease causation and are linked to the water, animal, and vector pathways, while others reveal significant knowledge gaps regarding febrile illnesses and associated risk factors such as the association of febrile illnesses with spirits and the reliance on ‘God’ as the only medicine. Of concern is that some of the health workers (who are also members of these communities) share these cultural/religious views regarding the etiology of these febrile illnesses and reveal an area for in-service capacity building, especially among health workers stationed in these frontier health units.

Additionally, these results contribute to the cultural understanding and characterization of febrile illness and its management in this region, as participants describe several forms of fever ranging from mild, acute, intermittent, and chronic fevers. They also elaborate on perceptions of febrile illness causation and the unique ability to distinguish different kinds of fevers using ethno etiological explanatory models in pastoralist, agro-pastoralist, and fishing communities at the front lines of these diseases. These theories of causation and association of fever with unique pathways, livelihood practices and seasons come from years of observation, and extensive folk knowledge about illnesses; they are also firmly held and influence their health seeking behavior. This influence of participant perception on health behavior reflected constructs of the health-belief model, where knowledge about the various forms of and susceptibility to febrile illness, and perceived benefits of, or barriers to treatment are important determinants of action/risk aversion (Table 3).

### Malarial fever

Malarial fever and its accompanying symptoms, and the resulting treatment sought was consistent with the biomedical model of disease causation and management in this study. Community members expressed greater confidence in the formal health system’s ability to address malarial fever as compared to non-malarial fever (although generally the health care system was considered impersonal and inefficient). This could be due to the extensive intervention and education programs targeting malaria in these communities, or the presence of rapid response teams at the village level trained to help in addressing malaria, (particularly childhood malaria through Integrated Community Case Management programs) and highlights the importance of community driven health interventions in such settings.

This study focused on areas of high febrile illness occurrence, and therefore requires further work in areas of low febrile illness to provide a comprehensive understanding of the current situation across this region. However, there was sufficient evidence that despite the advancements made in malaria control, pockets of hyper-endemicity due to tight coupling between human and natural factors still exist. Many times, these eco-syndemic connections increase the vulnerability (to diseases) of communities that are already poor with limited health infrastructure at their disposal. For example, the reported perennial malaria transmission along watering areas or lake shores compared to its bimodal pattern for the rest of the region. This natural factor combined with the need for water during the dry season increases the pool of susceptible individuals and thus disease transmission. Different agricultural land use strategies could also modify the occurrence of febrile illnesses as a result of increased suitable habitat for vector proliferation or increased vector-host interaction The epidemiological association of maize agriculture and malaria transmission has previously been defined and highlights the importance of a socio-ecological approach when designing health interventions in rural communities [67].

### Non-malarial Febrile illness

The availability of rapid malaria testing kits has revolutionized the diagnosis and treatment of malaria [11]. However, this does not account for cases of mixed infections or malaria negative fevers which often go mismanaged. It is clear from the community that causes of these fevers are diverse given the varying symptoms they experience and pathways they recognize. This differed slightly with previous work done in a similar context in Tanzania and highlights the importance of understanding context specific sociocultural factors that define the experience of illness in these rural communities [68]. Community members relied on ethno-etiological explanatory models to recognize and manage NMFIs, possibly due to the peripheral health system’s limited capacity to manage them and potential treatment failure experienced. While this pastoral/agropastoral model of health and illness reflected some biomedical conceptualizations as illustrated in Table 2, they were firmly rooted in a traditional view of health and illness that depicted humans (body, mind, and spirit) as highly connected to and influenced by their environment. These new models bear further study in terms of what diseases they elucidate- and how the management of these risks needs to be informed by such community perceptions of NMFIs.

The current WHO guidelines emphasize malaria treatment only on test positive results and discourage indiscriminate use of antimicrobials [1]. While important in addressing the issue of drug resistance and resource management, these guidelines seem to have produced an unintended consequence. Mistrust in the health system’s capacity to adequately address health challenges, especially those perceived to be non-malarial. This study highlights communities’ increased frustration with the formal health care system. Especially when definitive diagnosis for FIs is not provided or is delayed, affecting utilization of formal health care system even for presumed malaria cases among adults. This forces many to resort to self-treatment or herbal medicine and clearly illustrates that management of malaria cannot not be extricated from that of NMFIs in these frontier communities.

As previously noted, the narrowing of the conceptualization of malaria to parasitemia without recognizing the wider social construction of the disease reduces the efficacy of its management [69]. Thus, community members resort to alternative therapies based on their explanatory models of illness or delayed treatment seeking as has been previously reported in Tanzania [70]. Also of equal concern are the accounts of self-medication by community members frustrated by the health system’s ability to adequately address their health problems. This is prevalent in the East Africa region and highlights a worrying trend that requires urgent action in order to stem the rising antimicrobial resistance concerns in the region and other pernicious effects of self-medication such as masking of severe disease [70–72]. This perceived inefficiency of the health system has also produced a stoic culture that discourages individuals from seeking treatment for febrile illness, especially non-malarial illnesses. As a result, communities are trapped in a cycle of poverty exacerbated by chronic illness, reduced productivity, limited public health infrastructure and reduced livestock productivity due to the double impact of these zoonotic diseases on both humans and livestock [73]. However, local cultural explanatory models could help elucidate patient’s risk and thus inform treatment regimens at the peripheral health center. For example, in addition to the standard WHO IMAI guidelines, healthcare providers need to address a person’s location, livelihood practices, animal (domestic, peri-domestic, and wild) contact structure and ethno-medical practices to effectively manage their risk and prevent illness.

The ethno-medical approaches to management or prevention of febrile illness used could be protective or increase the risk of infection. For instance, the practice of boiling milk to prevent ‘milk fever’ was consistent with the biomedical explanation of ways to prevent milk borne illnesses such as brucellosis. Conversely, cow urine with its antibacterial, antifungal and antioxidant activities [74], could also be a potent pathway for transmission of pathogens such as *Leptospira* spp. The surge in these livestock associated and milk borne illnesses could be due to increased interaction between livestock and wildlife, and increased movement of pastoralists in search of pasture. Understanding the most important exposure pathways (animal associated) and spaces (watering areas) of disease transmission helps prioritize surveillance efforts and target limited diagnostic resources to areas of concern or pathogens of high suspicion based on community perceptions.

Our findings which unveil the health concerns linked to increased human livestock-wildlife interaction are consistent with previous studies that reveal increased human wildlife interaction increases the risk of conflict, injury and at times infection [22]. In addition to addressing the issue of rapid loss of forest cover and change in land use, the need for integrating communities into the work of conservation is beneficial for both communities and the surrounding wildlife [18].

Some practices although not widespread are common among subsets of the community members. For instance, the eating of raw liver by the hunters predisposes them to disease transmission-yet is considered as privileged status. An inverse relationship between the level of transformation of food and the social status tied to it has previously been postulated [75]. This would complicate efforts to address such an issue within the community and needs to be addressed as a risk factor for potential disease spillover from wild animals to humans.

## Conclusion

The results from this study summarize communities’ understanding of fever and their emic conceptualizations of key pathways of febrile illness in western Uganda. These observations have informed the development of culturally sensitive hypotheses about the etiology and pathways of fevers along a human-animal interface and paved the way for further research in this area. This information could also form the basis of community education, improvement of clinical algorithms and prioritization of disease categories/syndromes for diagnostic and surveillance investment. The water pathway was associated with the highest frequency and severity of febrile illnesses, acting both as a vehicle for pathogen transmission and a space that allows human animal interaction. Therefore, surveillance and prevention efforts need to consider the role of these water pathways when developing intervention strategies for NMFIs in regions of high human-animal interaction. Our results further reveal that health behaviors associated with febrile illnesses are greatly influenced by an individual’s cultural background, perception of the source and severity of the febrile illness and most importantly their perception of the health system’s ability to effectively identify the etiology and manage the severity of the illnesses. Health workers in areas of limited resources need to understand the variation in NMFI management across different rural areas or among different cultural/livelihood groups to effectively encourage health enhancing practices and mitigate against health lowering ones. Additionally, management and prevention strategies targeting infectious diseases need to be informed by this deep understanding of health influencing cultural and community perceptions to be effective.

Further research is needed in understanding strategies for improving; i) community members utilization of the health system, ii) NMFI treatment algorithms and iii) the link between conservation and public health in such settings. Improving community members’ utilization of the health system will require educating both the community and health care workers about effective ways of managing NMFI and danger signs to look out for in light of limited diagnostic facilities. Health messaging should intersect with and take advantage of the detailed empirical ethno-medical knowledge in communities. Additionally, developing communication channels between some of these informal health care providers with the formal health care delivery system could have the added benefit of facilitating early reporting of emerging illnesses or outbreak prone diseases. Furthermore, global health policies that seek to mitigate against drug misuse and resistance need to factor some of the unintended consequences of these policies that may emerge at the community level.

Given the complexity of NMFIs and its variation across different communities directed multi-sectorial approach that integrates participatory and bio medical techniques from different disciplines is required to effectively address NMFIs in areas of limited resources. Development of treatment algorithms, especially in peripheral health centers located in wildlife interface regions, needs to consider zoonotic illnesses as important differentials and the livelihood and cultural context of their patients. Finally, the potential role of conservation in the improvement of public health, or integration of public health and conservation approaches is worth further exploration in such communities that live adjacent to wildlife refuges.

This study has a couple of limitations. Given that it was conducted in areas of moderate-high malarial transmission. All results may not be generalizable across the entire population, however, can be transferred to areas of similar context. Additionally, the lower number of women participants in the focus groups conducted may have resulted in a limited exploration of the impact of gender on the risk and experience of febrile illnesses in this community.

## Supporting information

S1 Table Uganda Health Unit Levels, with Capacity Handled and Services Provided for each Unit. (DOCX)

S2 Table Illustrative quotes: Fever Etiology/Pathway. (DOCX)

S3 Table Selected Illustrative quotes from KIIs regarding febrile illness and human-animal contact. (DOCX)

S4 Table Emic Descriptions of Febrile Illness. (DOCX)

S1 Fig Average annual number of febrile illness cases presenting at health facilities within Sub-counties in Kasese District for 2012 and 2013. (DOCX)

S2 Fig Average annual number of febrile illness cases presenting at health facilities within Sub-counties in Hoima District for 2012 and 2013. (DOCX)

S3 Fig Photo highlighting human-animal interaction, livelihood practices and livestock production. (TIF)

S4 Fig Photo highlighting febrile illness risk pathway. (TIF)

S5 Fig Photo highlighting febrile illness risk pathway. (TIF)

S1 Text COREQ (COnsolidated criteria for REporting Qualitative research) Checklist. (DOCX)

S2 Text Coding Framework. (DOCX)

S3 Text Selected Participant Observation. (DOCX)

S4 Text Key Informant Interview Guide. (DOCX)

S5 Text Focus Group Discussion Guide. (DOCX)

S6 Text Focus Group Discussion Guide translated. (DOCX)

S7 Text Individual consent form. (DOCX)

S8 Text Individual consent form. (DOCX)
