# Supplementary material for: “There are many fevers”: Communities’ perception and management of Febrile illness and its relationship with human animal interactions in South-Western Uganda
Source: PLoS Negl Trop Dis. 2022 Feb 22;16(2):e0010125. doi: 10.1371/journal.pntd.0010125 (PMC8929701; doi:10.1371/journal.pntd.0010125)
Supplement: S1 Table — (DOCX) [file pntd.0010125.s001.docx]

S1_Table: Uganda Health Unit Levels, with Capacity Handled and Services provided for each Unit

| Administrative Level | Health Unit Level | Population Capacity | Services offered |
| --- | --- | --- | --- |
| Parish Local Council II | Health Center II | 5000 | “Preventative, promotive, and outpatient curative services, and emergency maternal deliveries |
| Sub-county Local Council III | Health Center II | 20,000 | All the above services. In addition, provides inpatient, maternal, and laboratory services |
| County-Local Council IV | Health Center IV | 100,000 | All the above services. In addition, provides emergency surgery, blood transfusion, laboratory services. Supervises level 2 and 3 |
| Sub-District | General Hospital | 500,000 | All the above services, but more comprehensive than HC 4. In addition, provides medicine, surgery, obstetrics, and gynecology, pediatrics, family medicine, and X-ray (plane and mobile) |
| District Local Council V | Regional Referral  Hospital | 2,000, 000 | All the above services. In addition, provides specialized services (Medicine, Surgery, Obstetrics, and Gynecology,  Pediatrics, ENT, Ophthalmology, Orthopedics, Anesthesia, Pathology, Psychiatry, Dentistry, and Community  Medicine. Have specialists, train nurses, have a blood bank, do basic and applied research and provide  Engineering services to facilities in its health zone. |
| National | National Referral  Hospital | 10,000,000 | All the above services, but more comprehensive and advanced than regional hospital. For instance, national  Hospitals offer advanced diagnostic services such as MRI and CT scans; they have super-specialists, and train doctors, pharmacists, dental surgeons, and graduate nurses and carry out advanced research.” |
