# Supplementary material for: “There are many fevers”: Communities’ perception and management of Febrile illness and its relationship with human animal interactions in South-Western Uganda
Source: PLoS Negl Trop Dis. 2022 Feb 22;16(2):e0010125. doi: 10.1371/journal.pntd.0010125 (PMC8929701; doi:10.1371/journal.pntd.0010125)
Supplement: S2 Table — (DOCX) [file pntd.0010125.s002.docx]

| Supplementary Table 2. Illustrative quotes: Fever Etiology/Pathway | |
| --- | --- |
| **Interviewer** | **Informants’ Comments** |
| Now tell us what are/could be the causes of the different types of fever?  What impact do you know of when these wild and domestic animals interact, including the impact on you- people?  What are the common illnesses that affect you in this place?  But now do you like meat from wild animals’ example antelopes?  In the past during hunting there was cultural beliefs/traditional thinking… do you know any?  We go to the second question, Do you know animals that we term as wild?  Do you interact with these wild animals  According to you what causes these buffaloes to appear during rainy season? | The major causes of malaria fever are linked to poverty. For example bush, a poor person cannot afford slashing, cannot afford putting up a good house. Other causes include bushes and stagnant water. FGD Male participant-Pastoralist community  The sanitation here is still poor. We do not see health personnel here. . FGD female participant-Pastoralists  We also have fevers all the time. Still connected to the use of water that is not hygienic…and presence of many flies in *Runga*...*Runga* is dirty... FGD female participant-Pastoralists/Fishing Village  For example the domestic animals like cows come with flies and small mosquitoes from the bush that bite us and we get sick of fever. . FGD Female participant-Pastoralists  Some of those animals come with cough and they end up transmitting it to us. . FGD Male participant-Pastoralists  Here we are living at the mercy of God why I am saying this is because the health center is usually empty of drugs you go there sometime they tell you no drugs no drugs! Some people end up dying so God is our most medicine. Anthrax we suspect is transmitted to our cattle by the wild animals and they die on the spot they don’t survive. . FGD Male participant-Pastoralists  When these small mosquitoes bite us we get fever and they usually have sharp teeth so we suffer a lot for living here, the park has many things that it brings. FGD Male participant-Pastoralists  There is competition between the duck and hens….you cannot have duck and fail to have chicken that are around…So we cannot have goats and fail to have cows. Right now people and animals are like friends…they live together-FGD female participant-Pastoralist community  We suffer from malaria so much because we are near the park and mosquitoes are very many in this place.. FGD female participant-Pastoralists  Cough is also killing us especially the men who stay with cows all the time we don’t know whether we get it from cows or milk. FGD female participant-Pastoralists  Ayaaaaa we suffer from malaria fever because we are bitten by mosquitoes in the lake and we don’t get enough treatment so we are ever sick… FGD Male Participant-Fishing Village  The major challenge these people have is that they sleep with their goats in the houses and the hens because they fear being stolen by thieves. These animals like goats, hens drink or eat on the same plates with people and those animals usually have diseases like cough this is why we are ever prone to diseases. FGD female participant -Fishing Village  Anthrax has also finished our animals we think it is transmitted by the wild animals because they share grass in the grazing field. FGD male participant -Pastoralist community.  Typhoid also as a result of unboiled water but the big number of people don’t boil \water and hence it’s not safe this leads to the outbreak of typhoid. FGD female participant -Pastoralist community.  Some people just think they are suffering from malaria whenever they feel bad but you find others have their other diseases like AIDS and they say its malaria. FGD female participant -Pastoralist community.  (Group) Ah ah ah haha we like it. We like it. We do eat. Unless it is someone’s totem. Even if it is killed here it will not reach the center here. It is more delicious than beef. Ha ha ha wild meat is very good and people like it. FGD participants Agropastoralist/hunting community  Yes hunting is associated with evil spirits (*embandwa*). These spirits help them (hunters) to kill these animals, without spirits it is very hard to get an animal and kill. FGD-Agropastoralist/Hunting  In the past such things were there, I also used to witness like tying a banana fiber around the stick or spear so that the curses of the dead animal do not cause febrile illness. But today people have received salvation so people just pray and get healed. FGD-Male participant Agropastoralist/ Hunting community.  In Bahima culture we are divided into two, the pastoralists and cultivators (crop). The cultivators do the hunting not the pastoralists, the pastoralists do not hunt. FGD-Male participant pastoralist community.  Even this water of the lake has caused diseases for example cough, malaria, you find a person without a single coin to buy good water of 100 shillings then they decide to drink lake water which is not safe then they end up sick. FGD Female participant-Fishing Village  The hygiene especially here the fishermen put their waste in the lake this is the same water we use at home so we can’t survive: FGD Male Participant-Fishing Village  We are largely affected by wild animals like crocodile, hippo even today the crocodile has eaten one man. Some diseases come but we don’t understand for example you feel pain in the stomach for long and you get fever , so we don’t know whether its water or something else or disease from wild animals- Participant-Fishing Village  Yes first of all our hygiene is poor especially *Alur* don’t have toilets. They use the bush instead of toilets, to prove this you go up there you will find that area is burnt completely- Participant-Fishing Village  Even if you have another disease we only mention malaria so we can’t go for malaria test so you find people talking of malaria yet we could be suffering from other diseases.  Malaria, back pain, Onchocerciasis , worms, chest pain and leg problems (sighs..) , flu, epilepsy/epileptic fits …it is so common as well among children… and is connected to fever, heart burn, (check this)-Female FGD participants -Agro-pastoralists  Let’s go with that but others have AIDS but they disguise that as malaria even when the person dies they will mention fever yet the person was killed by AIDS. FGD Participant-Fishing Village  In *our* health center they only check HIV/AIDS and (fever) malaria but not worms or others. If not they send you to the sub-county-Health center 3*.* FGD Participant-Fishing Village  If I take my cows for grazing and my cows get ticks (*engoha)* and you a person you take meat and milk from this cow you also get diseases [transmitted from these ticks]- . FGD-Male participant Pastoralist  *Brucella* is brought by milking cows immediately after spraying because the acaricide has not had time to take effect FGD-Female participant Pastoralist community  Our weather patterns have changed and it is because of our weather changes that we no longer have water and so we get diseases because we share our water sources with animals and wild animals which come to drink water… FGD-Male participant Pastoralist  Wildlife means those animals that don’t come near humans. They don’t want to reach near a human being. Those are animals which are not looked after and we believe they are the ones which carry diseases to our livestock because some eat grass like our animals. Such animals include baboons, monkeys, hare, buffaloes, hippos, fox, snakes, squirrels… and many others.  Oh yes we always interact with these animals in the fields, even in our homesteads-especially because we have fruit tress such as mangoes in our homesteads so this attracts them to our homes and we interact with them here as well… Now those animals for example monkeys, baboons, eat cassava therefore they always come. FGD Female participant -Agro-pastoralists  (Group response) Yes those ones come every day. We usually find them even in the gardens following the cassava and potatoes. They like these food items. FGD participants -Agro-pastoralists  We see them every day all of them …FGD participants -Agro-pastoralists  No, what about buffaloes? FGD participants -Agro-pastoralists  Except buffaloes which sometimes come during the wet season (April May June)-Pastoralist FGD  They run away because of tsetse flies. Another is the growth of vegetation/bush around where they can easily hide.  Generally the animal cannot forget where it once passed it will always come back. For example they used to camp at that hill. So there are pathways that these animals will always use from time to time year to year… |
