# Supplementary material for: “There are many fevers”: Communities’ perception and management of Febrile illness and its relationship with human animal interactions in South-Western Uganda
Source: PLoS Negl Trop Dis. 2022 Feb 22;16(2):e0010125. doi: 10.1371/journal.pntd.0010125 (PMC8929701; doi:10.1371/journal.pntd.0010125)
Supplement: S3 Table — (DOCX) [file pntd.0010125.s003.docx]

| Supplementary Table 3. Selected Illustrative quotes from Key Informant Interviews regarding febrile illness and human-animal contact | |
| --- | --- |
| **Interviewer** | **Informants’ Comments** |
| What is the major livelihood activity in this area  What health challenges do they face  Any other possible solutions  Any efforts to improve healthcare in some of these hard to reach areas?  Diagnosis of non-malaria febrile illness  What is the major livelihood activity in this area  What are the major health challenges here?  Are there diseases that people get from the animals?  Any efforts to improve health care access in hard to reach areas  Diagnostic services especially for non-malaria febrile illness?  Common illnesses in this place?  Are there any health impacts as a result of cattle keeping?  Have you had any complaint that some diseases are got from animals.  How about the health services offered are they of great help?  What kind of illnesses are common in this place?  What policy efforts have you put in place towards improving health access?  What is the major livelihood and major health challenges  Major livelihood activities and associated illnesses  Policy efforts towards improving health delivery and services in hard to reach areas  Have you heard about One Health  What are the livelihood activities of the different communities here?  What are the main livelihood activities in this area?  How can these challenges be solved? | **Sub-County Health Officer- Agro-pastoralist community**  They are mostly subsistence farmers (*balimi*) they grow maize, banana but we also have a few pastoralists, they don’t dig but their work is to graze cows  *Yah* they have diseases like TB because some of their cows which are usually sick so they get TB form these animals. They also put their (human) wastes anywhere and this increases the risks of strong diseases like cholera, so we forced them to dig pit latrines and use good water from boreholes, but they used to dispose of human waste anywhere resulting in increased diseases..  People have been educated on how to separate themselves from animals for example because of theft some people sleep with their animals in the same houses so this puts their lives in danger of getting animal diseases.  Even the pastoralists are advised to dig pit latrines because their human waste could mix up with animal waste which causes diseases this is common with the pastoralists of *Basongora*.  Others have constructed houses for their animals and this has reduced the spread of diseases from animals to people  In our sub-county there is no hard to reach areas we have the health centers in each community so if the drugs are here they come but when the drugs are not there they go to other health centers.  Absence of drugs influences people negatively because when they find no drugs they get discouraged and it changes their behavior negatively. People with serious malaria don’t come because they know that there are no drugs just the lab.  Now people used to come and collect drugs and keep them at their home or sell them but now when someone comes they test or examine you in order to give you drugs and if they don’t have the drugs they refer you to another health center.  There are drug shops and clinics also that some use especially for first aid while some resort to e herbal medicines and people believe in them so we have them “Uganda Ne dagala Lyayo”[local herbalist association].Others understand but others still believe in traditional healers and you don’t despise them…  **Local Council III Chairperson (Agro-pastoralist community )**  Usually these pastoralists have many cows for example I know of a person who has over 600 cows so they are hard to maintain all these cows especially giving them medicine even the land is small for them so you find them encroaching on peoples land or park.  We have buffaloes, elephants, lions, Uganda cobs, monkeys, rats so these are common ones. Someone had reared a monkey at his home it got used but I think after some time it went away  Right now there are no drugs in the health centers what I understand is that the drugs are brought but they are sold by officials. Other people use traditional herbals to cure diseases…  **Sub-county Health Officer-In-Charge (Fishing/Pastoralist Community)**  Zoonotic disease outbreaks in the community do occur and in order for people to stay safe after such an outbreak it’s the duty of the veterinary department and human health department to work hand in hand to treat and sensitize the people (victims).  Unfortunately this collaborative action has been challenged with the failure to hold occasional joint sensitization meetings resulting in a big absence of knowledge in addition to failure of identifying key personnel to lead these efforts. As a result community involvement and response has been weak and with little enforcement it’s hard to change or effectively influence people’s attitudes.  The major health challenges experienced are related to water shortage and their livelihood activities. Crocodile and hippo accidents often occur especially among the fishermen. Due to the fishing there are rampant movements which lead to family instabilities and violence. For the pastoralists brucellosis has been a problem however it’s not very common.  In response to these health challenges we have improved service delivery by establishing Health Center II and Health center III. However, equipment and staffing is still a challenge at these new centers. This in turn affects people attitude towards accessing and getting to these health facilities especially when they have to be referred even when they need simple tablets. We also do not have adequate number of laboratories, especially for febrile illnesses of zoonotic nature they lack different reagents and test kits.  Having not received the help desired most community members would go to private clinics instead of the recommended referral hospitals due to lack of funds most of which are working but not effective unless they are big and approved. Sensitization of the community (creation of awareness) is very important and so should be prioritized. It’s important to have recognized individuals from health related sectors in these meetings in order to create confidence in the audience to One Health workshops and seminars.  **Medical officer In charge Health Center IV-Pastoralist Community**  The primary febrile illness here is malaria. All treatment is guided by the rapid diagnostic test and blood smears. No treatment is allowed without malaria testing. We also have the capacity to diagnose other febrile conditions such as *Brucella*, *Hepatitis B* antigen test, HIV antibody test, *Syphilis* antibody test. We do not have the capacity to test for Typhoid fever and it normally is a diagnosis of exclusion. We encounter about 2-5 cases of Brucellosis every month and approximately 30 cases of fevers of unknown origin monthly. We neither test nor encounter other zoonotic diseases on a regular basis.  We have several challenges in the management of Brucellosis, for instance we are not actively looking out for it given the low suspicion index among clinicians, many times patients may resort to self-medication which may mask symptoms, also most of these people are mobile and so it is difficult to identify risk factors or exposures yet prevalence of the disease among the pastoralists is high. The long course of treatment (6 weeks of doxycycline + gentamycin) is hard for many to adhere to, so compliance is low. It is very hard to determine if the patient is cured following treatment.  **Health-in-charge Agro-pastoralist subcounty**  I don’t understand it very well but we have the veterinary doctors here they can explain more about that and then some animals have cough so we believe that people especially those who stay with animals all the time can get such cough. But I remember one time when they said we should be careful with animals because they usually have diseases especially the dead animals.  Here we have two health centers and they are located nearer to the community so this has helped people to access the health services but the only challenge we face is that most times people do not find the drugs and this makes them get a negative attitude so people’s behavior tend to be negative towards the health access because they usually find no drugs in the health centers  Usually blood samples are taken to understand what the patient is suffering from but sometimes the machines can fail to detect the illness and thus referred to other health centers .The health in charge does allocation of drugs depending on what the patient is suffering from  Then he allocates drugs depending on the diseases and the number of people suffering from the disease  Allocation usually disturbs people because others find it difficult to get the drugs of the diseases they are suffering from.  **Female clinical officer Pastoralist community**  Diarrhea is common here at least we get a victim weekly. Then fever is also common here because some people do not boil their water for drinking .The containers where they fetch water from are also dirty so they find themselves drinking dirty water hence getting fever…  I am not sure but I think when they reach the park they drink water stagnant that is shared by animals so they end up getting diseases in that water such as cough, febrile illnesses…  I have not heard anyone but they usually share water with animals so the chances are high that they may get diseases from the animals. Others take unboiled milk so I don’t doubt that some diseases are really got from animals. For example some people tend to sleep with the animals in the same house and this is very dangerous so people are sensitized about the dangers of staying with animals in the same house.  More to that the mosquitoes breed form the bushes around the homes so people have been advised to clear the bushes around them.  Not at all as you can see this place is very new and inside there is nothing like a thermometer we are here just seated but we don’t really have anything to give to the patients we refer them to other health centers. There is malaria test kit, I even borrow money to pay the cleaner of this place.  **Local Council III Chairperson Agro-pastoralist community**  Yeah, there was the issue of anthrax which killed many national park animals and it is a threat to the domestic animals. The warthogs sometimes when people take them they contract diseases but we are not sure of the diseases so it has a negative impact because when such animals die it means the health status of the animals is not good.  Common diseases include malaria then some diarrhea, as we said HIV and sometimes cholera in X and Y parish – but malaria is the major cause of illness in this place according to the report we get from the medical records.  The services are not enough although our health center III is supposed to admit patients but it does not so that malaria is not healed in early stages so it makes them admitted to hospitals, but also people were given mosquito nets but they are not using them otherwise it would be a forgotten story. People don’t want to change their attitude. Safe water is not there in fact 65% of the people drink unsafe water.  Yes about drugs they come and get finished before another consignment comes. We have emphasized that we come up with latrines and sensitizing the masses. The problem is quite challenging e.g. bringing drugs on time is a role of government but we also have a role to inform the government sometimes we enforce the law by even using the police for these people to understand certain situations. For example when there is an outbreak all stake holders are put on board for mobilizing village health teams go and sensitize the community, political heads are there and others  **Senior Veterinary Officer Pastoralist Community**  The major livelihood activities in this area revolve around agriculture in the form of agriculture and livestock keeping as well as trade (in the agricultural products). Most health impacts are as a results of wildlife and livestock interaction during grazing times yet the livestock is not well segregated from humans (owners) and hence there’s the possibility of zoonotic disease crossing over (wild to domestic and vice versa) and in some cases man is caught up in between.  Unfortunately some of the cultural beliefs among the people further complicate this situation. For example Kanyampara believe that anthrax cannot kill them yet their companions *Basongora* believe that anthrax (*kakoto*) kills and have been careful in addition to taking precautionary measures. The Kanyampara people do not burry anthrax corpse in addition to eating dead wild animals. A most effective way to reduce this phenomenon is sensitization effectively on radio, however it requires money for airtime.  **Administrative Officer-Pastoralist Community**  As mentioned the existence of Rwenzori and Queen Elizabeth national parks in between which Kasese is located and in Queen Elizabeth there is fishing, hunting, collecting firewood and during some encounters in the park people tend to contract zoonotic diseases such as anthrax during free interaction. And in fishing villages there are diseases like bilharzia and communicable diseases like cholera especially due to poor sanitation. There is a lot of cross border movement of animals from Queen Elizabeth N.P to Virunga Park without supervision especially at the border.There is need for collaboration between Uganda and Congo in order to address the cross border movements, thus controlling likely health outcomes  There is a plan to construct staff quarters in areas like landing sites such that medics can attend to people fully when there is need, this will move hand in hand with extending referral hospitals. In addition there is an effort and plan of collaborating with wildlife and veterinary doctors since they are the people in charge of wildlife hence need to organize joint meetings to sensitize people and other relevant persons. About clinical diagnostics of illnesses of zoonotic nature (not malaria) there’s a big gap because the areas at the periphery of the park do not have labs that are strategic.  In some areas people believe that they have been bewitched in times of outbreaks e.g in katwe during the cholera outbreak.  **Senior Agricultural Officer Agro pastoralist Community**  Major livelihoods are agriculture based, mostly crop agriculture. About 10% rely on cattle as a source of livelihood. Most individuals own about 1-2 cows while others have more than 20. Piggery is gaining popularity and we have about 10-15% of household relying on the pig enterprise. Pigs are often responsible for dispersal of some of these infectious diseases and parasites (since they are mostly allowed to roam at best they are tethered in gardens and not held in holding pens). Most cattle are found along the lake shores and these communities rely on milk as a source of food and if they need to change their diet they buy from the stores. A typical farmer is a poor one, and so their animals may lodge in their homes increasing the possibility of disease transmission from livestock to humans  We have the wildlife game reserve-have chimps, baboons and monkeys…people do come in touch with this wildlife because of the encroachment and degradation of the environment. For example we have a chimp that picked a child from the garden….If managed well we can improve the area and make it a tourist attraction. We have some controlled hunting areas as well near the lake-possibility of ecotourism exists…Interaction is also there with the livestock especially in these controlled hunting areas. Dogs are often a species that interact with these wild animals. The chimp-human conflict requires attention, they like forested areas but because of degradation they are pushing into people’s gardens. There is need to develop systems for joint surveillance to facilitate also joint action when need arises…Communities need to be involved as active stakeholders and participants in this process as well. Their input in accurately defining the problems and its multifaceted nature is invaluable…  Our community is a society that welcomes many people and this has health implication…These people can be a problem….We get people from neighboring countries. Diseases are spread often during this movement. Cholera has been spread like this and also Ebola from Kibale to Hoima..…Conflict is also present when these individuals move into the community. They often rent land for a period of 1-2yrs land and soon wrangles emerge…Water points is also a common area for the exchange of diseases. At these water points there is also wrangling for land and water.  **Local Council-3 Chairman-Agro-pastoralist community:**  Yes I have. We were taught once in a meeting when people were being educated about diseases transmitted from animals to humans. This training helped because people did not know diseases were spread from animal meat.  In this community about 70% are crop farmers and about 15% are animal farmers while the rest do other activities. *Banyakole* are the herdsmen and they like concentrated milk. Therefore they take raw milk and yet we were taught that this leads to diseases. When a cow aborts I have witnessed the pastoralists consume the fetus and they say it is delicious. Yet you may not know why the cow aborted and this spreads diseases…They are fond of carrying out delivery with their bare hands this may cause disease as well. Small scale crop farmers also use bare hands when working in the fields, without any boots. This too may cause disease.  **Health Officer –In charge of Health Sub-District (Pastoralist/Fishing /Hunting Community**  The main activities in this area are agriculture livestock and fishing. Although crop agriculture does not have many negative health impacts, sometimes people encroach unto wet lands and forests for cultivation and as such get exposed to more disease causing organisms. While among the fishing communities many are always near stagnant water which serves as a great breeding ground for mosquitoes…the poor soils are not good for latrine construction and result in cholera outbreaks. We also have interaction of humans and wildlife in various ways that result in disease transmission: during hunting, during grazing livestock in the park or bushes, also at home with wild animals such as rats that live in the house but also venture into the wild and could result in outbreaks such as plague…Dogs also interact with wildlife (fox) during hunting or eat carcasses of wild animals by doing so transmit diseases.  We need to sensitize all stakeholders at all levels of administration and emphasize the importance of this collaboration approach (One Health). This also needs to involve the community. For example we have the Village Health Teams (VHTs) who are involved in testing and treatment of malaria, they also support our surveillance activities including quick reporting to the department of health if sudden mass deaths of animals is observed. Yet they do not connect with the Vet department. |
