# Supplementary material for: “There are many fevers”: Communities’ perception and management of Febrile illness and its relationship with human animal interactions in South-Western Uganda
Source: PLoS Negl Trop Dis. 2022 Feb 22;16(2):e0010125. doi: 10.1371/journal.pntd.0010125 (PMC8929701; doi:10.1371/journal.pntd.0010125)
Supplement: S4 Table — (DOCX) [file pntd.0010125.s004.docx]

**Supplementary Table 4: Emic Descriptions of Febrile Illness**

| Local Description | Medical Term | Local clinical signs | Local etiology | Local Treatment | Cultural group | Medical diagnosis and treatment based on described signs |
| --- | --- | --- | --- | --- | --- | --- |
| *Omuswijja we kisararu* | Onchocerciasis | Rash, fever, *okuhuma* (blindness) | Black flies (*Biramfunzi)* | *Omugina* (*Asteraceae spps*), *Aloe vera* | Agro pastoralists | Onchocerciasis |
| *Omuswijja gwe byenda* | Diarrhea (enteric fevers) | Fever, headaches abdominal pain, diarrhea, joint pains | Contaminated water and/or milk, poverty, change in diet from non-dairy to dairy as the seasons change from dry to rainy season | Urine and dung; local herbs.  Milk fresh milk and mix with fresh urine/dung-causes one to diarrhea and pass out the fever… *Omushumba gurwe gwamatee*,  *Orutotomia* (Lamiaceae spps) | Pastoralists and Agro-pastoralists | Malaria and/or Typhoid |
| *Omuswijja gwe mibu* | Malaria | Headache *(Omutuwe)*  Fevers, joint pains, muscle aches, convulsions in children,  *Omuliro* (elevated body temperatures) O*kunyaara* yellow (yellow urine), *ahamaisho ga hinduka* yellow (icterus) | Mosquitoes, bushy areas, poverty, stagnant water | Warm fresh cattle urine  *Ekibirizi* (*venoniya spps.)*  *Omuko* (*Erythrina tomentosa)* *Enkokorutanga (*Aloe vera)  Orwihura Momordica foetida Schumach. | Pastoralist, agro-pastoralist, fishing and hunting communities | Malaria |
| *Omuswijja* | Blanket term potentially referring to; Malaria, Typhoid fever, Yellow fever, viral respiratory infections | Headaches, Acute fevers, Abdominal pain *(ekirwaire),* body pain. Cough/difficulty breathing | Exposure to mosquitoes while fishing, drinking contaminated water while fishing, drinking water contaminated by wild animal urine | *Ombirizi* | Fishing community/ Pastoralists | Malaria |
| *Esinyinga ya amyi* | Influenza | Cough, headaches and high fevers | Dry season, sharing living spaces with poultry | Urine and dung.  Orwihura , Momordica foetida schumach. | Pastoralists/Fishing village | ARI or Malaria |
| *Omuswijja gwe nte* | Brucellosis | headaches, joint aches, prolonged fevers, back aches, stiffness, rigors (*okukankana*) | Raw Milk and milk products such as *Shabwe*  We also buy fresh milk from our pastoralist brothers and if we do not boil the milk well we end up contracting this disease drinking traditional fermented milk  More in women due to production and use of raw milk products | Urine, *Aloe vera* (pastoralists)  Seek medical treatment for chronic fevers | Pastoralists/Agro-pastoralists | Malaria/Brucellosis |
| *Omuswijja gwe bisolo* | Livestock associated zoonotic febrile illness | Fever, elevated body temperatures *(omuliro)* muscle aches, joint aches, back aches, stiffness of neck (*Omuraramo*), | Raw milk (*amata*), undercooked meat,  Flies, mosquitoes and ticks (*engoha*) from livestock | *Ombirizi* | Pastoralists | Malaria |
| *Omuswijja* | Zoonotic diseases associated with hunting, slaughtering and handling wildlife | Acute fevers | Malignant spirits from the wild when hunting is done without adhering to needed cultural ablations | Currently resort to prayer | Agro-pastoralist/Hunting community | Malaria |
| *Omuswijja gwa mazi* | Typhoid dysentery/Cholera? | Headache, diarrhea, abdominal pain, fever, acute illness | Poverty, Poor hygiene and sanitation, Lack of Latrines, Flies | Dung and hot water, (*ebussa gwe nte*)  *Aloe vera* | Pastoralist/Fishing communities | Typhoid dysentery  Cholera |
| *Ekimerero* | Unknown | *Okutungura* (feverish), Acute piercing pain (*Okuchumuta*) in ones sides/ribs (*orubazo*), heart(*omutima*), (back), malaise, joint pains, failure to bend, pain in neck- | Linked to contact with livestock | Unknown | Pastoralists | Malaria |
| *Ekihinzi* | Flu, head cold | Feverish-body is hot, body aches, malaise, bronchitis | Seasonal, livestock linked, seen in cows and goats, seen mainly among herdsmen, | *Ombirizi* | Mainly Pastoralists and cattle traders | Acute Respiratory Infections (ARI) |
| Omuswijja gwe njoka we munda | Helminthiasis | Abdominal pains, common among children | *Njoka* (worms) | *Orutotomia, Ngusuru* | Pastoralists, Agropastoralist, fishermen/hunter | Helminthiasis |
| Omuswijja obutakira (fever that never heals) | Unknown | Persistent fever without diagnosis | Unknown, suspected to be connected to their livestock rearing | *Rukaka* (*Aloe vera*) | Pastoralists | Malaria treatment |
| Omusiijja gwe ekifuba  (nkororo) | Tuberculosis | Fever, Chest pains, persistent cough | Dry seasons, sharing cigarettes, Sharing alcohol, raw milk | Aloe vera, warm fresh urine | Pastoralists | TB |
| Entunuka | Paronychia | Cellulitis |  | *Aloe vera* |  | Paronychia |
| *Omuswijja gwe msimbu* | Neurocysticercosis?? | convulsions | Eating pork undercooked pork, | Believe alcohol destroys the infective larval stages | Agro pastoralists | Malaria? |
| *Omuswijja gwe obusheru.* | Measles | *Omuswijja* (fever), *Kasesa* (rash) | Dry Season | *Ombirizi* | Agropastoralists/ Pastoralists/Fishing village | Measles |
| *Akazimba* | Anthrax | Fever (*omuswijja*),  *Ekifuva* (cough/chest pains) | Contact with infected livestock or consumption of undercooked contaminated meat |  | Pastoralists | Anthrax |
