# Supplementary material for: “There are many fevers”: Communities’ perception and management of Febrile illness and its relationship with human animal interactions in South-Western Uganda
Source: PLoS Negl Trop Dis. 2022 Feb 22;16(2):e0010125. doi: 10.1371/journal.pntd.0010125 (PMC8929701; doi:10.1371/journal.pntd.0010125)
Supplement: S2 Fig — (DOCX) [file pntd.0010125.s006.docx]

Supplementary Figure 2: Average annual number of febrile Illness cases presenting at health facilities within sub-counties in Hoima District for 2012 and 2013
