# Supplementary figures and images for: “There are many fevers”: Communities’ perception and management of Febrile illness and its relationship with human animal interactions in South-Western Uganda
Source: PLoS Negl Trop Dis. 2022 Feb 22;16(2):e0010125. doi: 10.1371/journal.pntd.0010125 (PMC8929701; doi:10.1371/journal.pntd.0010125)

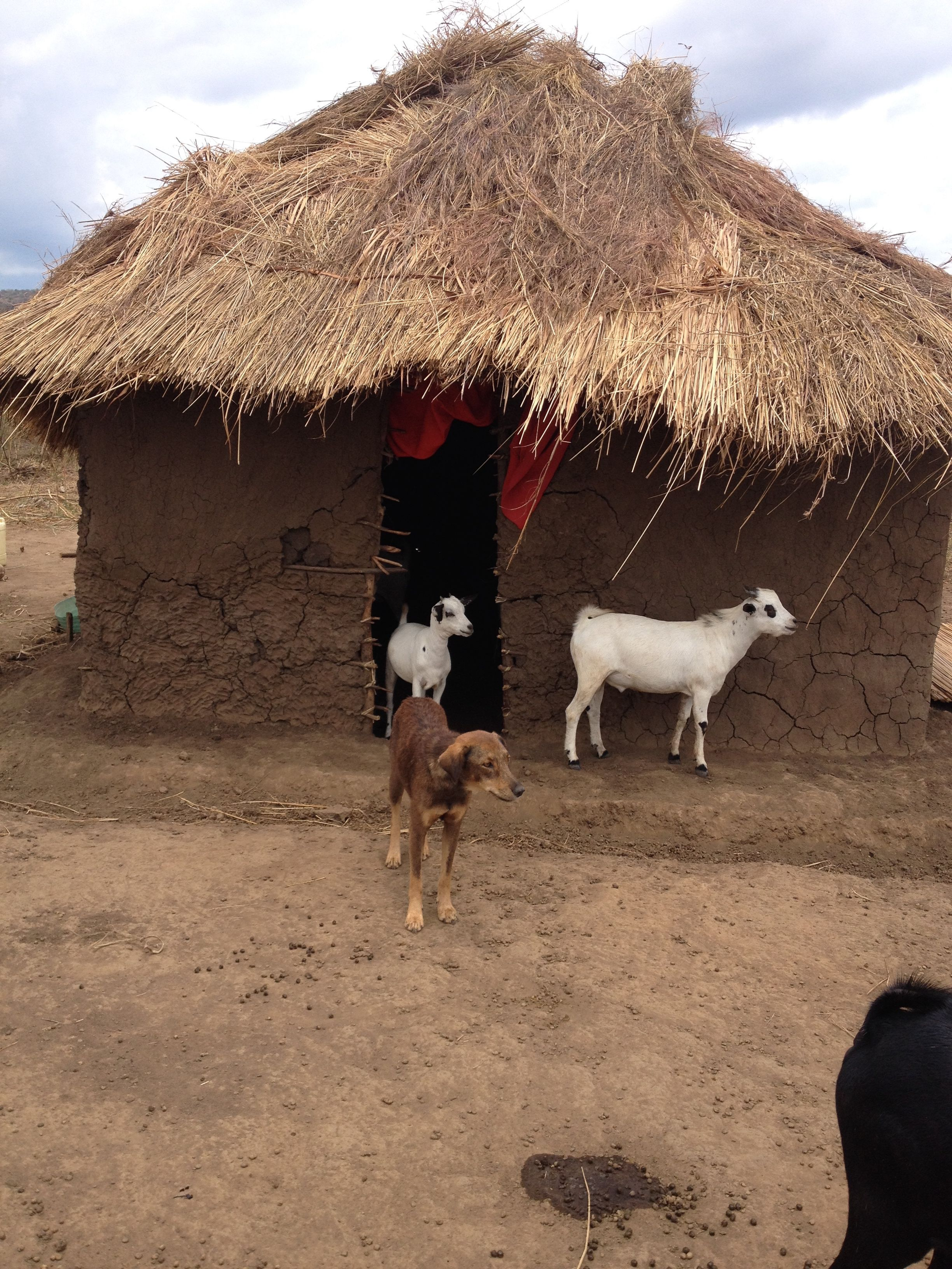

Supplement: S3 Fig — (TIF) [file pntd.0010125.s007.tif]

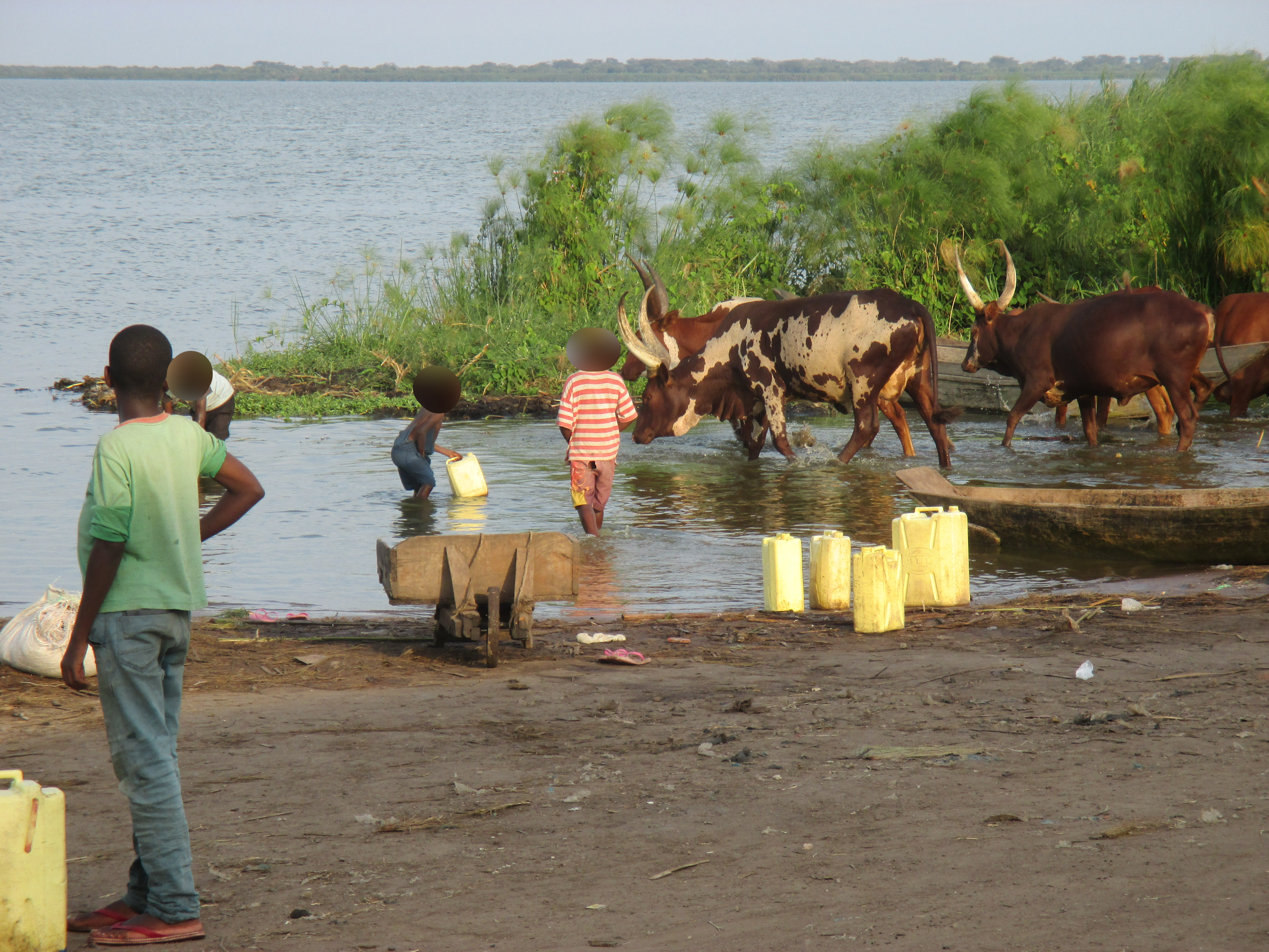

Supplement: S4 Fig — (TIF) [file pntd.0010125.s008.tif]

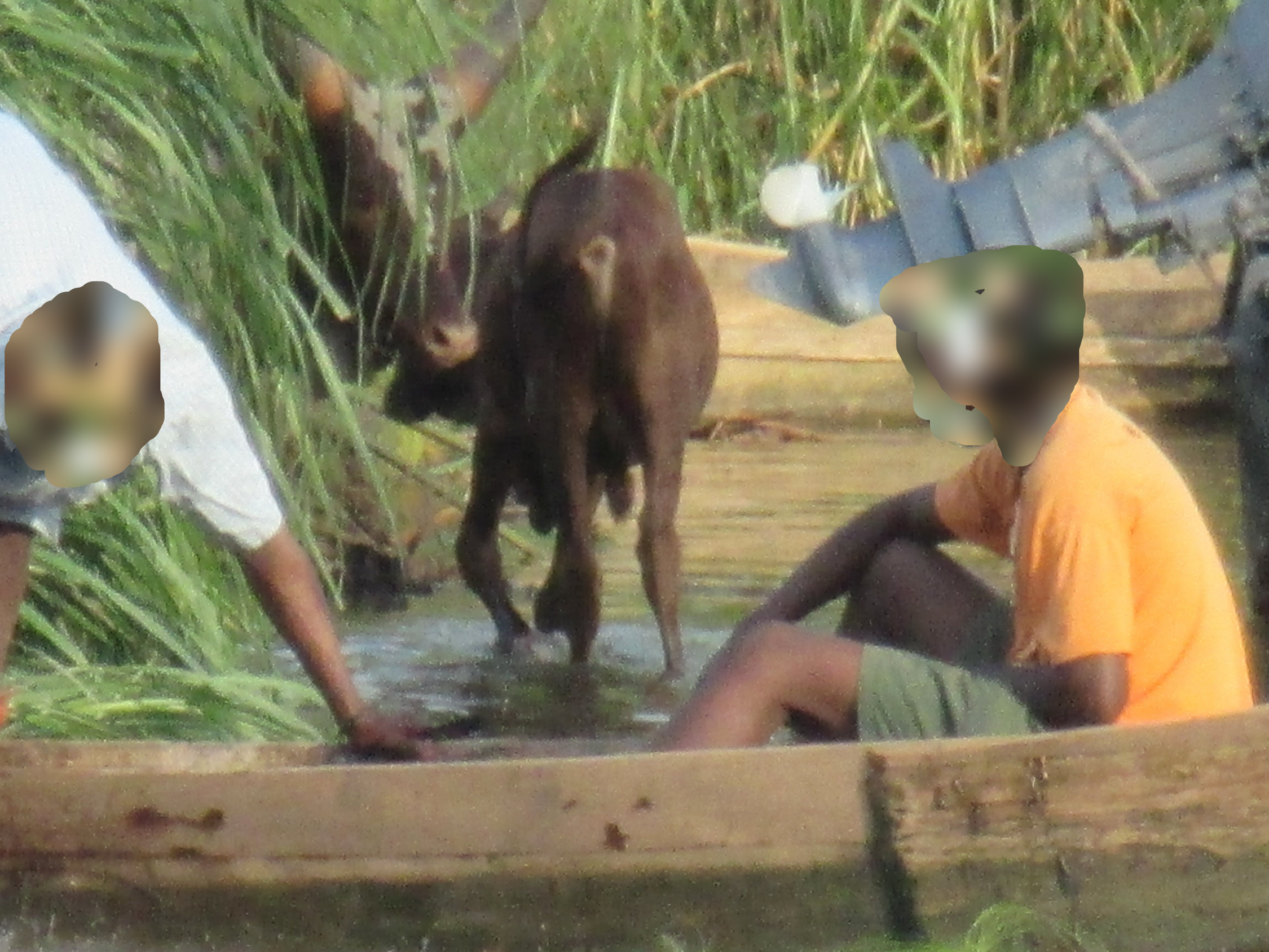

Supplement: S5 Fig — (TIF) [file pntd.0010125.s009.tif]
