# Supplementary material for: “There are many fevers”: Communities’ perception and management of Febrile illness and its relationship with human animal interactions in South-Western Uganda
Source: PLoS Negl Trop Dis. 2022 Feb 22;16(2):e0010125. doi: 10.1371/journal.pntd.0010125 (PMC8929701; doi:10.1371/journal.pntd.0010125)
Supplement: S2 Text — (DOCX) [file pntd.0010125.s011.docx]

| **Short description** | **Details** | **Inclusion** | **Exclusion** | **Examples** | **Not this…** | **Log of changes** |
| --- | --- | --- | --- | --- | --- | --- |
| Code 1 | | | | | |  |
| Adaptation | An evolution of practices and perception to cope with emerging challenges or evolving times | All practices and perception developed to cope with evolving times and communities | Diversity of action or perception across different communities | Because more regulations have come in, No killing of wild animals.  Another one, people have a lot to do. Instead of going to hunt the whole day you would rather go and buy a kilogram of meat in the market | Hunting is like our taboo even the park authorities know it very well that we do not eat any meat from wild animals | *(captures iterative edits made by team to arrive at consensus on final code*) |
| Livelihood Strategies | Different techniques employed by community members to make a living or sustain their families | Includes the strategies and unique cultural drivers/determinants of livelihood-strategy choice  Highlights unique impacts of livelihood strategy on health outcomes | Does not include cultural activities engaged in for leisure e.g. hunting as sport | For us we are pastoralists. We rear cattle, goats and others have poultry. But indeed for us our income comes from only cattle. We do not realize much income from other livestock. | Most especially hunting was like a game so people use to go and play that game. Even during Christmas season people used to kill warthogs. Pigs, buffaloes, Uganda cobs. |  |
| Code 2 | | | | | | |
| Agro ecological pathway | An attribution of febrile illness occurrence to surrounding agroecosystem | All agricultural practices, systems and environments that are linked to febrile illness occurrence | Increased risk in particular communities due to unique hygiene or socio cultural practices | During maize season people get severe malaria, during rainy seasons we experience malaria cases due to breeding of mosquitoes  OR  We suffer from malaria so much because we are near the park and mosquitoes are very many in this place | Yes, but also in our homes there is poor sanitation. There is rubbish which is not disposed off and stagnant water |  |
| Code 3 | | | | | | |
| Agro pastoralists/pastoralist | Distinction between different cultural livelihood strategies | All cultural preferences and practices associated or defining pastoralists and agro pastoralists | Other non- livestock associated livelihood strategies even if agriculture is involved | “We crow/cultivate cotton, simsim, beans, maize, tomatoes”  OR  “For us we are pastoralists. We rear cattle, goats and others have poultry. But indeed for us our income comes from only cattle. We do not realize much income from other livestock” | Fishing is our major source of income especially me “Jimmy” I rely solely on fishing to cater for my needs and md family |  |
| Code 4 | | | | | | |
| Animal Diseases | Description of types, frequency and season of animal disease occurrence | All pathological occurrence observed or diagnosed by animal health professional | Does not include human specific diseases with no observed or diagnosed link to animal disease occurrence | ….there is a disease that attacks goats. The goats swell here we don’t know that disease. It usually kills goats  OR  There is anaplasmosis, abortions but here we suspect that abortions are brought about by pesticides, herbicides sprayed on cotton. They usually die during that period | …Yes it is the same especially measles, cholera etc. here cholera usually likes visitors. A visitor cannot survive |  |
| Code 5 | | | | | | |
| Bush Meat | Cultural preferences guiding the consumption of bush meat | Cultural preferences or taboos that govern the acquisition, and consumption of bush meat | Does not include perceptions and practices around hunting | …But we *Bahima* according to our culture nobody is supposed to eat meat from any wild animal even if those eaten by other people like buffaloes. | …Another thing people are educated they don’t want hunting |  |
| Code 6 | | | | | | |
| Hunting | Cultural or socioeconomic preferences guiding the hunting of wild animals | Factors that push or prevent community members from hunting | Does not include perceptions and practices around bush meat consumption | No the population of wild animals is decreasing and even the law does not allow once you are caught they will arrest you | No no no “*ngaaha*” all those animals listed are eaten. One of the tribes took a baboon from me and ate it… |  |
| Code 7 |  |  |  |  |  |  |
| Animal link/Interaction | Communities different types of animal interactions (domestic and wild) | Captures the different direct and indirect pathways of interaction between humans and animals and the potential risks or benefits of this interaction | Does not capture non-animal linked pathways e.g increased febrile illness during maize season.. | We share lake water with animals because wild animals drink or stay in it even domestic animals like cows, goats, sheep drink this water so you find them all gathered at the lake | Ayaaaaa we suffer from malaria fever because we are eaten by mosquitoes in the lake and we don’t get enough treatment so we are ever sick. |  |
| Code 8 |  |  |  |  |  |  |
| Human Illness | Description of all forms of human health challenges in community | Types, causes, frequency, experience, of human illness caused by biological, physical or chemical agents | Non-Human Illness (illness that are specific to animals-unlike zoonotic diseases) | Here we have many diseases like Malaria, back pain, Onchocerciasis , worms, chest pain and leg problems (sighs..) , flu, epileptic fits …it is so common as well among children… and is connected to fever, heart burn, | Animal diseases include,east coast fever, cowdriosis, worms and others. |  |
| Human Febrile Illness | Communities explanation of febrile illness | All explanations of nature, names, causes and categories of febrile illness | All causes and categories of non-febrile illnesses | *“There are many fevers*”  All febrile causing illnesses are called “o*muswijja”* (fevers). |  |  |
| Febrile Illness Management | Communities explanation of how they manage febrile illness | All descriptions of the different ways in which they manage fever and rationale behind their choice of fever management strategies | All strategies of managing non-febrile illnesses | “Urine also cures cough [and fever] for example you drink urine in the morning when the cow has just urinated and you take two cups you get healed” Female FGD Pastoralist community | “Dung also heals broken legs and arms.”  “Cow gee also is used for healing broken legs and arms you keep on pressing the leg with this gee and it gets healed.” |  |
