# Supplementary material for: “There are many fevers”: Communities’ perception and management of Febrile illness and its relationship with human animal interactions in South-Western Uganda
Source: PLoS Negl Trop Dis. 2022 Feb 22;16(2):e0010125. doi: 10.1371/journal.pntd.0010125 (PMC8929701; doi:10.1371/journal.pntd.0010125)
Supplement: S3 Text — (DOCX) [file pntd.0010125.s012.docx]

Selected Field Notes from Participant Observation of Selected Areas in Hoima and Kasese District.

Fishing/Pastoralist Village:

The place is clearly a fishing village the smell of fish is unmistakable and rents the air hitting me in the face as we descend down the escarpment to the shores of *Runga* which are dotted with mud huts and grass thatched roofs…Its clearly a vibrant place home to many foreign passersby. There was loud music that gave the place a perpetual carnival-feel not sure why or what but there is something transient about this community, they seem to live for now... Maybe it’s just me with my etic lens and maybe my hunch is true…since the area seems to be as much a home to many as it is a corridor to many more who are funneling in and out of the Lake shores of Lake Albert looking for humanitarian refuge or simply interested in eking a living. Our arrival caused a bit of a stir, having arrived in a government pick up whose driver was willing to risk the treacherous drive down the *Waki* escarpments. This is where a large population of the pastoralists live, the perpetual supply of fresh water, flat river banks and undulating plains that roll into thickets and wildlife dotted bush and forest is a big attraction for them. An attraction that ensures their large herds of cattle have a good supply of water and feed throughout the year.

We park on higher ground about half a mile from the strip of densely populated human settlement along the shores of Lake Albert. We then slowly make our way down to the little town of Runga, carefully descending ridges and little gulleys. The land seems to be constantly battling the chiseling force of fast flowing water as it drains from the escarpments into the lake. The people are friendly. Having left our vehicle we easily blend-in blurting out greetings in the local Lunyoro language and waving pleasantly to those we encounter. There are some permanent structures, some modified pit latrines created to accommodate the sandy soils of the area, and many poorly constructed pit latrines. No wander these communities are constantly battling cholera and other water borne disease outbreaks...Small ruminants, mainly goats are seen dotting the landscape, of course the occasional duck and hen also welcome us to Runga. The homesteads are lined up in neat rows most are mud walls and grass thatched roofs with a few brick walled and tin roofed houses. We are soon at the local Runga trading center. Our abode for the next few days is a local lodge that has tiny rooms with brick walls and tin roofs, no running water but we do have some electricity. We soon settle in and whether the water was running or not, the hot bath was a welcome relief. Water is a precious commodity…clean water that is. A major challenge, in addition to the sanitation problems they seem to have, is clean running water. I am glad we stocked up on our supply of fresh clean drinking water.

A stroll down the market center reveals somewhat of a diverse community, different cultural groups blended in this community. As I hang out at the market place I hear Swahili speakers with a blend of French words-most certainly neighbors from across Lake Albert’s shores. Congolese who took the brave move of crossing the lake from the DRC often in little boats some of them unable to swim. From what I gather some of these attempts are tragic… As I ask around and mingle with the masses I realize that many of these individuals are fleeing a life of trauma, many are women and children-brave to tackle the treacherous journey across the deceptively calm fresh waters of the approximately 2000square miles of Lake Albert. Some of these are migrant workers all in pursuit of economic opportunities without formal citizenship status. There is a lot of buying and selling, I walk past many little shops, small dispensaries with community nurses or village health team members running them, open air restaurants, women frying fish, milk being transported in plastic containers, all shrouded in the local blast of some carnival music. Lots of young men huddled in small groups, I am told these are fishermen taking it easy waiting for the night fall when they venture out into the dark and silent waters of Lake Albert. Many of those I encounter are *Alurs*, a Nilotic ethnic group who originate from the northwestern side of Uganda and are part of the larger Luo group. The *Lugbara* who also are part of the Luo community are present in large numbers. Historically these communities have relied on fish as a source of protein and fishing as their major livelihood. This explains the migration from the West Nile region to these shores in pursuit of livelihood opportunities. I also encounter the indigenous *Banyoro, Bagungu* (a blend between the *Alur* and *Banyoro* and are mainly fishermen and small scale traders) and the migrant *Banyankole* who are from the pastoralist community. The *Banykole* are not many here in *Runga* it looks like most of them live in a nearby village called *Waaki*, some 3 miles away from the *Runga* trading center. They are easy to spot most have a shepherd’s rod they call *nkoni*…its both functional and symbolic, symbolic of their pastoralist status that they are proud to claim and pronounce. It is amazing that this seemingly isolated community has a vibrant and amazing cultural blend all very evident as I stroll down the trading center. I wonder how these multiple cultures affect their perception of illnesses and thus their health behaviors. I wonder how these cultures fuse and blend, perhaps it all revolves around the deep desire to survive… Fish and Cows that is what seems to be keeping this micro economy booming. We soon join in the buying and selling, after a brief negotiation, with a local restaurateur (passionate middle aged woman running her open air fish joint) we squat unto tiny little stools and wait for our freshly fried fish. She also quickly prepares a bowl of beans for us. “These are very expensive here” she quips. See, nothing much grows in these fishing villages, the soils, climate, heat, rainfall pattern and lifestyle makes crop agriculture a rarity. Therefore most of these are ferried in from the upland agricultural communities. *Runga* is known as one of the vibrant fishing villages in the region, it now makes sense why there is a perpetual carnival spirit. There is lots of fishing, buying and selling of all manner of goods some legal others illegal… fueled by the constant migration of people. We soon gobble up the fresh fish, bowl of beans and a cassava based stiff porridge…and call it a night…what a day it has been!

The early morning call to worship reminds me that this is truly a melting pot with different cultural and religious groups. I step into the piercing chill of the morning breeze, determined to appreciate a different aspect of this community. It is soon evident that there other sub terrain activities…a group of young men pass by with spears in their hand-it looks like a hunting party. This soon explains the large and visible presence of dogs in this fishing village...moving in noisy packs…looks like they are useful for both security and also for hunting. Some of them have lots of mange…others could do with a bath …interesting community-never a dull a moment! Soon meet another group of young men…as I walk towards the lake, the gentle breeze is refreshing and the lake’s waters gently lap unto the shores. Young men are hauling in their catch. Indeed fishing is big in this community. Looks like they are some of the young men I met earlier on, who ‘rest’ during the day and work at night. What a dangerous line of work, may be this also contributes to the ‘live for now’ attitude I seemed to pick on. Still see other fishermen on boats in the lake busy setting up their nets perhaps having finished with the nights activity. I ask the local elder about the hunting, and he concurs it happens and the game meat is quite popular-some even opting to eat some of the juicy pieces raw... He explained how some wild animals like buffalo that stray into the community are usually hunted and meat distributed, while the skin is discarded in the lake. He further added that some claim to hunt baboons to feed their dogs, yet those who lack dogs also partake of this meat… *“Where do they take the meat? ”* he wondered. Maybe some ends up on the bad street (a local open air food market known for its variety of meats…of known and unknown origin).Heat is rising the sun is now out in all its brilliance…it’s time to stroll back to the trading center and get away from the heat that seems to be steadily rising.

There are lots of ducks and pigs. Some wallowing by the road side, while others were in people’s houses. Seems many are comfortable having their livestock inside their homes. Maybe out of a concern for thieves, wild animals or the marauding pack of dogs. Nets are strewn all over…being spread outside and repaired by fishermen, women drying sardines in the sun, lots and lots of sardines…called by the locals- *mukene*. A big truck by the roadside …looks like these are traders buying dried fish/sardines along these fishing villages…truly a lot of buying and selling …fish and more fish… I meet a young man who seems to understand both Swahili and Luganda, languages I am fairly comfortable with. So we soon strike a conversation and I join him in his day’s activity…he runs a motorcycle business, I offer to pay for his services as he takes me round the fishing village, and soon we are on our way to the next village…*Waki* where lots of pastoralists live. It’s an informative time with him, he is an affable fellow who seems to mingle effortlessly with community members, he is skillful at his work. Riding these machines along sandy soils that a riddled with gulleys is no mean achievement. He seems to enjoy his work and talks of the brisk business by the shores of *Runga* and *Waki*. He has been able to build a small enterprise from scratch and now has a team of other cyclists he works with…lots of outsiders who come in to buy fish, lots of money during peak fish seasons, lots of challenges due to the poor infrastructure and road network. Limited health personnel, the escarpment seems to pose yet another major barrier to accessing timely health interventions. We soon part ways I make my way to *Waki* and yet another chapter of the many sides of this community seem to open before me. It’s a community faced with myriad challenges yet resilient and determined to use their available resources to try and beat the odds. From the sound of it, sometimes they win and sometimes the odds are against them…

*Waki* is just like *Runga*, lots of buying and selling, cosmopolitan fishing hamlet. Loud music in the background playing incessantly, small restaurants, lots of ducks, pigs and dogs. Found a place selling duck meat…its terribly hot. Lots of herdsmen (*balalo*) their sight unmistakable donning hats, stick in hand and sometimes a shawl to help fend off the early morning chill as they take the herds out to graze.

I make my way to the watering points by the lake. Soon I realize why it’s the land of fish and cows (and goats). Large herds of cows descend unto the shores. It is a beautiful sight to behold. They call this *kushera*, it’s a time for the herds men to get together. It is cultural for herd owners to join in this activity when possible. Allowing them to tend to their herds as the animals water and get a reprieve from the hot afternoon sun. Most of the herdsmen and cattle owners are from the *Banyankole* tribe, every man stick in hand. I join them as they water their cows. The presence of a vet is most welcomed…their passion for this trade is evident. To them this is more than a trade it’s a way of life…it is a calling of sorts…Most of the cattle are indigenous breeds-long horn *Ankole* cattle. There are some Friesian crosses, but these are few and are not favored due to their low disease resistance. They look at their herds with admiration know their cows by name-it’s amazing! Soon the watering point is filled by a few hundred animals from different herds all comingled. I do not seem to notice any pattern or schedule in the watering process almost seems its first come first served with as many animals gently lapping on the lakes’ waters. There is little concern for potential disease transmission between the herds. Perhaps they all know each other and are aware of who is who…probably also know of where disease emerges from and whose herd is affected. They seem to have a network of herdsmen; it feels like a close knit community. The *balalo* are skillful with their animals, calling, whistling and corralling their cows with what appears to be effortless skill. This is the time to identify any weak, sick or injured animals-they complain of abortion in their herds. There are several goats as well but not as many as the cows. The *balalo’s* respect for their animals is because it confers on them a sense of identity, is a major source of food –most of which would be dairy based and where possible made from raw milk, they also rely on their animals as one would on their savings. I now see why fish and cows make this micro-economy go round.

The livestock management is extensive, the pastoralists move with large herds of cattle for miles on end looking for pasture and water. The plains by the lake shore are great for grazing. Some of the herds came trickling down the escarpments so it is evident that they move as far as the upland areas and possibly into some of the surrounding forest reserves in search of pasture.

One section has cattle watering; boats parked with fishermen working on their equipment, and women and children fetching water while others are also getting a reprieve from the harsh afternoon heat-bathing or swimming. If the trading centers are melting pots of cultures, these shores are a mixing point of humans, animals and perhaps pathogens as well. Quite an interesting and very telling sight, the need for water pushes both man and beast to the brink…

Trading Center and Community life in Agro-pastoralist/Pastoralist border region:

Participant observation involved visits to the trading center in *this border* town and walks through the community. We interacted with village leaders as we visited different communities within the sub county to get an emic view of the villages and some of the salient features. This sub county is a border community that lies along Lake Albert. It is densely populated with a great mix of cultural groups given its location. It receives several visitors, many of them cross the lake from the neighboring countries such as the Democratic Republic of Congo (DRC). This was evident given the multiple languages spoken in this area including Swahili which is not commonly spoken in Uganda and less so among the Western communities of the country. Given the larger more dynamic population there seems to be more activity, the trading center is filled with people moving up and about buying and selling. Everyone seems busy, eager to eke a living. The area seems to be densely populated; more people, plenty of small-scale agriculture, saw individuals in the field using traditional/subsistence farming methods. Individual land ownership seems to be the major land tenure system here. There seems to be more economic activity here compared to other sub counties visited thus far and lots of young people. There is plenty of rain. Lots of people walking along the road. There seems to be a lot of unutilized parcels of land and plenty of bushy areas. Several wild rodent sightings. Water is obtained from communal taps-mostly in yellow plastic containers; there are plenty of vibrant small trading centers and several houses under construction. Most of the houses in this area are constructed using brick, motor and tin roof. Several water collecting points are visible as one travels down the main roads-these are open ponds found in low lying areas and used for washing cars, bicycles, motorcycles and agricultural activities. Lots of motor cycles at these washing areas...Saw commercial nurseries-looked like tree seedlings…young men seemed to be working on these sites. These washing areas are full of activity and are normally near bushy wooded area or swampy regions were open shelters are also constructed for workers. It looks like some brick making is also going on and some of these individuals are involved in it. Lots of bush clearing in some sections…some forested areas seem to be cleared out as well. Looks like there is increased demand for land and settlement, looks like most of this is fueled by the influx of migrants and increased economic activity and agricultural activity in this region. Local leaders talk of conflict over land given the influx of people to this region. This conflict pits the pastoralists (who move are mobile) against the agro-pastoralist over land and grazing areas. Most immigrants from Congo settle by the lake’s shores and are attracted by the promise of peace and an opportunity to make money given the vibrant economy in the region. Most small scale farmers produce vegetables, cereals and have an average of about 3 acres of land. A few large scale farmers exist in this area and mainly produce cotton and tobacco. Milk is mainly produced by pastoralists and sold to consumers directly. While live animals are sold to butchers and they sell the meat to consumers. Small scale famers also sell some of the extra produce to nearby markets where middlemen buy and distribute to Hoima, Kampala and Juba. Several primary schools sighted. Pass by a road side pork joint that seems to serve some alcohol as well…several men and women move in and out of this pork joint. It is made of wooden poles the sides are propped up by polythene material and the structures have tin roofs…the men and women here seem to be as giddy as pupils.

Community members seek medical care from both government and private health centers, but mostly private because of the distance from the community to the health center III or IV. Sometimes the personnel at the health center are stretched and thus services limited. Some attend traditional healers while others visit religious leaders for prayers. Traditional healers are well known and often enumerated alongside the existing formal health infrastructure by local leaders we encountered. Many local leaders were not aware of the One Health collaborative effort between various health professionals prior to our outreach to these areas. Most request for more health centers that are better equipped and a better supply of vets and vet drug shops in the area. There seems to be some constrain in accessing these animal and human health services at the community level. Visited the sub-county headquarters, several local government offices, and even more activity, managed to go to the sub county health center iv. Health officer in charge of the health center and outlined their capacity and catchment areas they serve. This facility serves 2 other sub-counties and also caters for individuals from a neighboring district. The place is busy with many patients quietly waiting in the waiting area that is very well ventilated. Mostly mothers with their infants and toddlers here for vaccination or some form of treatment. The silence is often punctuated by a crying baby who is growing impatient, feeling unwell or scared of the health care providers. Several health care workers are busy shuttling from one patient to the next. There is a nearby lab that has several lab technicians who are busy working on samples and peering through microscopes. The health officer in-charge revealed that most of their febrile cases are malaria. They receive about 2-5 Brucellosis cases (seen mainly among mobile individuals who move/travel a lot) monthly, sometimes self-medication by the patients, masks presentation. He further explains that adherence of treatment among patients is sometimes hard given long treatment course. Although they have testing capacity for brucellosis, sometimes, the suspicion index among clinicians for these non-malarial febrile zoonotic infections is low. The health center has some testing capacity that includes malaria antigen testing and microscopy, hepatitis antigen testing, syphilis screening, TB culture and sensitivity, HIV-viral antibody test and Brucella antibody test (IgM). Lab does not have the capacity to test for typhoid. This is primarily a diagnosis of exclusion. Medical records are tracked and then digitized and shared with district data team to be inco-operated into the Ministry of health HMS health record systems. Health center has some outreach activities into the communities these activities are focused on; immunization, health education-reproductive health, community health work especially focused on water borne illnesses, and village health teams/Aids community volunteers

Agro-pastoralist Sub-county headquarters

Lots of motorcycles called *boda boda*, bicycles also used for transportation. There is also quite a lot of construction of houses, several primary and secondary schools sighted, even a small community college as well. Students are evidently present in this area. The agricultural activity in this area is higher with more large scale production in this community. Most farmers produce cassava, maize, sorghum on individual plots of land. Homesteads have fences and are closely packed together. Young boys and girls were picking firewood. There are several river beds, and papyrus swamps that are used as car washing bays. Several heavy trucks seen along the road, maybe picking agricultural produce for distribution outside the district. Tea estate also present…large scale production with a fully flagged commercial hub focused on processing the tea leaves for distribution and subsequent value addition.

The trading center has several general merchandise shops, several mosques and churches, a metal work shop that is indicative of the high level of construction work in the area, several restaurants, several roadside open air food joints and even a local dairy that collects milk from farmers and distributes it to consumers. Customers were seen carrying milk in plastic bags-this looks like fresh raw milk perhaps distributed by a roadside vendor or the local milk distributor. We pass by several community members on their way back to their homes from the trading center. One particular lady stands out…she is carried on a boda boda clutching her child and several live chicken at the back of the motorcycle.

Visited a local famer with a mud wall and grass thatched kitchen, main house is brick walled and has iron sheets-she has ducks as well as chicken-that have no visible structure-probably live in house. Has goats as well that are tethered near the open air kitchen. She has several children who are in school-one just finished her primary education and is expecting a good result. Her husband was not in but available. She talked of brucellosis affecting several people in her area and she focused on her afternoon chores. We discussed animal production, questions about her farm and animals kept popping up. She was grateful to have us visit with her and excited to show us her agricultural enterprise.

- Area has small herds of dairy cattle that are mainly Friesian or Jersey crosses. There seems to be quite a number of small holder dairy farmers in the area. Individuals keep one or two cows. Visited the Health Center IV and the in-charge revealed that the center serves a catchment area of about 15,000 people most of whom are referrals from the lower health centers or self-referrals. It’s a large hospital with situated on several acres of land, not as busy as what we encountered at the health center iv in the agro pastoralist/pastoralist border region. All services are free and the febrile illnesses often encountered are UTIs, TB, Brucella, respiratory tract infections, Malaria and viral infections. Standard workup for febrile illness:
  - Urinalysis for Urinary tract infections
  - Rapid slide test or blood smears for malaria
  - Sputum analysis for TB
  - Management is decided upon by **clinical judgment + lab diagnosis**

All patients present with a complaint of fever and about 20% are fevers of unknown origin. In a week distribution of febrile illnesses: Difficult to estimate, but his guess was:

- - - 4% malaria
    - 60% Upper respiratory tract infections
    - 8-10% Urinary tract infections
    - 20-30% Undiagnosed

Most treatment is syndromic and lab diagnosis is primarily; Brucellosis antibody test, Malaria test smears, Typhoid widal test. He confirmed they have about 2 cases of Brucellosis a month and the biggest challenge patients are faced with is the long course of treatment so adherence is low and relapse not uncommon.

Agro-pastoralist Farming Community:

This is clearly an agricultural community. Several small trading centers and secondary school encountered as we make our way into the community. Spotted men with timber on bicycle, several brick walled houses a few mud houses with grass thatched roofs. Lots of construction going on, it looks like community members have access to money, or there is an influx of migrants into the area. Area is bushy with small access roads. The soil looks rich, the temperatures cooler, the place greener than most of the pastoralists’ communities visited earlier. There are lots of trees and the area seems to receive plenty of rain. The homesteads are organized in a linear fashion side by side along the access road some of them have paddocks for grazing their cattle, while others tie their animals to a pole or tree that allows access to pasture or that allows them to cut and carry feed to the animals. The herds are larger than those kept in other neighboring communities we passed. A number of Farmers keep more than 10 dairy animals using a paddocking system. It’s a pleasant sight, it looks well organized and the pastures look well kept. Larger heads of cattle. Lots of pasture, lots of well-organized paddocks…Fewer people in the trading centers looks like most of the activity is on these farms, everyone is busy with their parcel of land and herd of cattle. The crop production is small scale and subsistence. There is a lot of small scale dairy production-I am happy to experience a different cattle production system and appreciate some different perspectives.

We visited a woman who had a dairy cow and several pigs. We joined her as she tended to her animals. The pigs were in very good condition and one had several piglets…The dairy cow had cataracts probably the effects of ocular larval migrans or chronic infection with heamo-parasites. Not much can be done to the eye but the animal can be taken care of well and will still produce. We discuss options and she soon complained of lots of deaths among dairy cows in the area…sounds like it may be some tick borne infections and so she requested for more training on how to raise her dairy cow.. She further revealed of a trend she has observed where farmers slaughter ill animals to save on protein loss…but shortly after strange syndromes are observed among the people who consumed this meat…what could be the problem she asks…we explain the concept of zoonotic disease, some cardinal examples and danger signs to watch for e.g. when anthrax is suspected. Her cow is tethered while her the pigs are allowed to leisurely roam around the compound. We also talk about brucellosis…she has heard of it. Her brick house with tin roof is hard to miss and is quite a contrast to the open air kitchen which is basically a grass thatched open shelter supported by wooden poles. She allows animal access to this area…and seems to have started on the evenings meal given the pot of food quietly simmering over open fire.

We swiftly make our way to another farmer, the sun is receding and evening chill is setting in. The farmer is a woman with a healthy looking herd of dairy animals. She has several cows, they are probably 20 or more, all in very good shape. She welcomes us with glee and is excited to see us. She knows we are vets and is eager to share her experience and seems to have a list of questions lined up for us. There are several structures in the homestead. Some brick walled others made of mad and plastered using cement. Her paddocks are well organized and a few of the cows have recently calved. The calves bay for their mother as they spring across the paddocks. It is a beautiful sight to behold. At the very end of her farm is a cattle crush, most of the other producers did not have it. She seems to be invested in her dairy enterprise and is in it for some money not just subsistence. Her herd is made of some *Boran* cross, *Friesian* cross and Guernsey cross. Some goats are also present roaming freely and interacting with about 3 or 4 dogs on her farm and a couple of pigs are also present; the animals , including the cows, freely mix with each other. The dogs are allowed to roam all over her farm, they seem to be her security plan. We join her as she inspects her herd and take note of a newly born calf with swollen salivary glands. We examine the calf, most probably an infected salivary gland-we discuss options and advise on what needs to be done. There are several young boys and girls probably her children or her relatives or workers children. They are excited to help. After about an hour or more we make our way out.

Next we visit a home with a semi-intensive piggery unit. The farmer uses rain water for home use. Animals have a built shelter and situated less than 20meters from the main house. Main house was made of brick and motor. The piggery unit is made of brick and tin roof, one is able to access and inspect the animals without coming in contact with the pigs. The famer seems to be keen about biosecurity guidelines. The animals are the large white breed and seem to be doing very well. Looks like business is brisk for the farmer. Farmer also practiced crop agriculture as is the case with most of farmers in this area. Yard well-kept and has some dogs on property-dogs allowed to roam freely in the home and neighborhood. Kept mainly for security purposes…

Pastoralist Community

Expansive land that appears communally owned. Welcomed to elders homestead where a number of pastoralist men and women are gathered. Although the cows are probably out grazing it is clear that these pastoralists keep large herds of cattle. There are several goats in the yard, left behind to be attended to by the women and children. The smell of milk is unmistakable, milk cans visible, some washed and placed on a makeshift dish rack to dry in the sun. The morning breeze briefly interrupts the rising temperatures. The elder has a well-kept home, its brick walled building with a tin roof. The floor is cemented and there are several chairs and large wooden table at th center of the room and a wooden shelf with a radio at one extreme corner. There are lots of flies, everyone seems comfortable, I guess it’s because of the milk and cattle. The home is neat and clean, we all have to remove our shoes as a rule as we enter the house. The elders seat, after which young men and women seat in a semi-circular fashion around the room. The youth seem progressive-some of their leaders are women. Women seat together on one side of the room, young men lift their hands to speak as we consult with this community regarding their experience with animal health challenges. Soon we are served a warm glass of milk…food does not seem to be a problem. The community is extremely warm and welcoming. Their knowledge is deep in matters livestock production. They are primarily from the *Bahima* tribe and seem to have great sense of camaraderie and consensus. Tick borne disease seem to be a challenge for their livestock, they manage these with local herbs. Water is a problem so they boil their water to avoid water borne diseases. They seem to be fairly health conscious. Recognize illness using body temperature and clinical signs such as jaundice. Also use the local health center to diagnose fevers among them. However the health center sometimes does not have drugs so rely on larger referral health facilities at the district headquarters or go all the way to the capital city seeking medical help.
