# Supplementary material for: “There are many fevers”: Communities’ perception and management of Febrile illness and its relationship with human animal interactions in South-Western Uganda
Source: PLoS Negl Trop Dis. 2022 Feb 22;16(2):e0010125. doi: 10.1371/journal.pntd.0010125 (PMC8929701; doi:10.1371/journal.pntd.0010125)
Supplement: S4 Text — (DOCX) [file pntd.0010125.s013.docx]

**Key Informant Interview Guide**:

1. **Are you aware of the One Health approach? What is its relevance to your current position and duties?**
2. **What are the major livelihood activities in this region? Probe health impacts and possible solutions?**
3. **What is the perceived impact of current health and animal health disease control policies?**
4. **What is the role of wildlife in this community-opportunities and challenges? Probe:**
   1. Policy efforts to address human wildlife conflict
   2. Ask about wildlife of interest from a community health perspective-rodents, primates, bats, potential pet-wildlife interaction
5. **Evolution of hunting in this community the risks and opportunities. Probe:**
   1. **Policies restricting or controlling the practices…**
   2. Its drivers and possible impacts from a community development perspective
   3. Any known/documented or perceived health impacts of this practice?
6. **Describe inter-sectoral** collaborations for zoonotic disease surveillance and control. Probe:
   1. Existing gaps and potential solutions
   2. Community involvement in the surveillance and control of these zoonotic diseases.
7. **What are the policy efforts currently in place towards improving health care access especially in hard to reach areas and what array of issues influence communities’ health care seeking behavior?**
   1. Probe clinical, diagnostic and chemotherapeutic services especially for non-malarial febrile illness of zoonotic nature.
   2. Alternatives commonly sought and health beliefs surrounding this phenomenon.
   3. Opinions regarding policy recommendations and implementation of integrated approach to community health (One Health)
8. **Do we have any migration into and out of this community?**
   1. Policy Management of mobile populations both from a human and animal health perspective
   2. Current management of any conflicts; perceived causes and potential solutions
