# Supplementary material for: “There are many fevers”: Communities’ perception and management of Febrile illness and its relationship with human animal interactions in South-Western Uganda
Source: PLoS Negl Trop Dis. 2022 Feb 22;16(2):e0010125. doi: 10.1371/journal.pntd.0010125 (PMC8929701; doi:10.1371/journal.pntd.0010125)
Supplement: S5 Text — (DOCX) [file pntd.0010125.s014.docx]

Focus Group Discussion Guide:

1. What are your major livelihood activities in this region? **Probe:** why and how?
2. Perception of wildlife. How would you define wildlife?
   1. Describe the kind of human, animal, wildlife interaction that exists in this community. **Probe:** nature, frequency and place of interaction (prode both park and lake). Ask about wildlife of interest-rodents, primates, bats, potential pet-wildlife interaction
3. Previously in our African communities hunting was a crucial part of the society. What was the situation in this community and how has it evolved over the years?

**Probe:** Involvement in hunting? Kind of meat obtained from this activity? Consumption in the community? What commercial activities are tied to hunting? Any other complimentary activities that go hand in hand with hunting?

1. Describe the human and animal illnesses present in your community and how the community deals with them. **Probe:** source, risk factors, seasonality, most commonly experienced illness/syndrome, duration, associations, transmission, impact on community, understanding of fevers/febrile illness and types of fevers, traditional beliefs surrounding disease occurrence, coping mechanisms, **community based management (what local strategies would they/do they employ)**
2. What kind of health care support is available for community members and what array of issues influence their health care seeking behavior? Probe clinical, diagnostic and chemotherapeutic services. Alternatives commonly sought and health beliefs surrounding this phenomenon.
3. What kind of movement of people do we have into and out of this community? Are there any observations that are linked to this movement? Probe migration and displacement and its impact on health in the region…
