# Supplementary material for: “There are many fevers”: Communities’ perception and management of Febrile illness and its relationship with human animal interactions in South-Western Uganda
Source: PLoS Negl Trop Dis. 2022 Feb 22;16(2):e0010125. doi: 10.1371/journal.pntd.0010125 (PMC8929701; doi:10.1371/journal.pntd.0010125)
Supplement: S6 Text — (DOCX) [file pntd.0010125.s015.docx]

Ebikaguzo bya hamu nkekitebe

1. Mirimo ki mukira kukora eri yokubemerezaho omukicweka kinu? Kaguliriza habwaki kandi bagikora bata?
2. Balinda enkooko, empunu ne embuzi. Barara nabyo omumaju gabu muno muno ekiro rundiki haha?
3. Bakozesa ebintu nka enkali yebisoro kutamba endwaire zo rususu omubantu?
4. Kirungi oyetegereze bika ki ebyebisoro ebibakira kusibika omumaka gabu omukicweka eki.

2. Entwara ye bisooro byomukisaka. Babitwara bata omukicweka?

a. Bisoro ki ebyekisaka, ebyomuka ebikwikara nabantu omukicweka? Kaguza obwingi bwabyo, mirundi eingaha bija omubantu kandi omubicweka byankaha omukyaro omu. Kaguza ebisoro ebyomukisaka nka kawamuje, ebitera, embugubugu, kandi embwa hamu nenjango biterana.

3. Omumyaka yeira okuhiga kikaba kikuru muno omu Afrika. Kikaba kita kunu? Kandi mpiduka hinduka ki ezinyakubaireho omu myaka erabireho omu kicweka kinu? Okuhiga kunu kukaba kuta kandi bisoro ki mwakiraga kwita kandi nyama ki eyebisoro mwalyaga. Byobusubuzi ki ebyali omukuhiga. Mirimo ki end nkokuhakura enjoki, okuhaiga endali eyabaaga nayo eri nkokuhiga…….

4. Ndwara ki ezikwata abantu rundi ebisoro omukicweka kinu kandi mukora muta kuzijanja rundi kuzetangira? Biki ebiziretereza,zijanjanjara zita,zikanya kasumi ki omumwaka, zibarabya zita, kandi abantu obuzicwekaho batekereza bata kandi mukora muta kuzimaraho omukicweka kyanyu….

5. Byobwomeezi ki rundi bujanjabi ki obukuhebwa omukiceka kinu kandi abantu babweyambisa bata? Kaguza bajanjaba, bakebera ebintu nko musahi rundi emibazi egabwa haali abarwaire. Haraho obujanjabi obundi bwona abantu bakozesa omukicweka kandi enyikiriza omubyobujanjabi eri eta?......

6. Abantu bagenda omubicweka bindi rundi abantu abandi baija nibabungira okuruga omubicweka ebindi. Haraho ekintu kyona kyakabagaho habwokulibatana kunu . Kaguliriza obu araba hakabagaho ekintu kyona habwokufurufuruka kwabantu omukiceka eki…..
