# Supplementary material for: “There are many fevers”: Communities’ perception and management of Febrile illness and its relationship with human animal interactions in South-Western Uganda
Source: PLoS Negl Trop Dis. 2022 Feb 22;16(2):e0010125. doi: 10.1371/journal.pntd.0010125 (PMC8929701; doi:10.1371/journal.pntd.0010125)
Supplement: S7 Text — (DOCX) [file pntd.0010125.s016.docx]

You are invited to participate in a research study of human diseases that are associated with febrile illness in Hoima, Uganda. You were randomly selected as a possible participant because you are a resident of the study area. We ask that you read or listen to all that is contained in this form and ask any questions you may have before agreeing to be in the study.

This study is being conducted by Dr. Michael Mahero of the University of Minnesota’s Department of Veterinary Population Medicine in collaboration with Makerere University, College of Veterinary Medicine, Animal Resources and Biosecurity, OHCEA (One Health Central and East Africa) Ecohealth Project. Support for this project is from the NIH Fogarty International Center’s Fogarty Fellowship through the Northern Pacific Global Health Research Fellows Training Consortium.

**Study Purpose**

The purpose of the study is intended to investigate the spatial distribution (community spread) of acute febrile disease syndromes in relation to level of urbanicity (municipal/local council size and level of development) and agroecological zones, and identify livelihood practices that influence exposure to potential zoonotic and (re)emerging diseases.

**Voluntary Nature of Study**

Participation is completely voluntary, and that my decision will in no way affect the quality of future medical or animal health interventions that I receive

**Study Procedure**

If you agree to participate in this study, we would ask you to do the following:

1. To respond to an investigator administered questionnaire.

**Risk of Study Participation**

The study has the following risks.

- Disclosure of personal information

**Benefits of Study Participation**

- There are no direct benefits to study participation.

**Alternatives to Study Participation.**

Not participating in the study

**Study Costs/Compensation**

No compensation for study participants.

**Research Related Injury**

No foreseeable study related injury to study participants.

**Confidentiality.**

The questionnaire responses of this study will be kept private. In any publications or presentations, we will not include any information that will make it possible to identify you as a subject. Your responses for the study may, however, be reviewed by representatives from the

departments at the University of Minnesota with appropriate regulatory oversight.

**Contacts and Questions**

If you have any questions or concerns regarding the study and would like to talk to someone other than the researcher(s), you are encouraged to contact the Research Subjects’ Advocate line D-528 Mayo, 420 Delaware Street S.E., Minneapolis, Minnesota, 55455; telephone (612) 625-1650.’ OR JCRC IRB Offices Plot 101 Lubowa off Entebbe Road. Telephone +256414201148.

**Statement of Consent**

I have read/listened to the above information. I have asked questions and have received answers. I consent to participate in the study. My consent is indicated by signing, my thumb print or drawing an “X” in the box below.
