# Supplementary material for: “There are many fevers”: Communities’ perception and management of Febrile illness and its relationship with human animal interactions in South-Western Uganda
Source: PLoS Negl Trop Dis. 2022 Feb 22;16(2):e0010125. doi: 10.1371/journal.pntd.0010125 (PMC8929701; doi:10.1371/journal.pntd.0010125)
Supplement: S8 Text — (DOCX) [file pntd.0010125.s017.docx]

You are invited to participate in a research study of infectious diseases that are associated with febrile illness in Hoima, Kasese and Fort-portal, Uganda. You were randomly selected as a possible participant because you are a resident of the study area. We ask that you read or listen to all that is contained in this form and ask any questions you may have before agreeing to be in the study.

This study is being conducted by Dr Michael Mahero of the University of Minnesota’s Department of Veterinary Population Medicine in collaboration with Makerere University, College of Veterinary Medicine, Animal Resources and Biosecurity, Conservation for Ecosystem Health Alliance-CEHA, Hoima District Local Government and One Health East and Central Africa –EcoHealth Project. Support for this project is from the NIH Fogarty International Center’s Fogarty Fellowship through the Northern Pacific Global Health Research Fellows Training Consortium.

**Study Purpose**

The purpose of the study is intended to investigate the spatial distribution (community spread) of non-malarial febrile disease syndromes in relation to level of urbanicity and agroecological zones, and identify livelihood practices that influence exposure to potential zoonotic and (re)emerging diseases

**Study Procedure**

To help with this research, we would like to have a focus group discussion about the following topics: Wild animals, health, livelihood practices such as farming and livestock rearing, human-animal interaction and your association with the environment both domestic and forested areas. The discussion should take about one to two hours to complete

The answers of all the study participants will be taped, transcribed and combined together into a report, and we believe that this information may help others better understand the situation in this area.

**Risk/Benefit Statement**

There are no direct benefits or risks to you for participating in this study. We do not offer any payment for participating in this discussion. The discussion should be completed in approximately 90-120 minutes.

**Voluntary Nature of Study**

Your participation in this discussion is completely voluntary. You do not have to participate in this study if you do not want to. You may stop participating and withdraw from the study at any time without penalty. You do not have to answer any question that you do not wish to answer or discuss. If you have any questions about this research, or about your rights as a participant, we will be happy to try to answer them.

**Privacy Statement**

We will make every effort to protect your privacy. We will write down your answers to the questions we ask, but we will not keep your names with the notes we take during the interview, and we will not use your names in any reports that we write about this research. However anything you say in this group interview could be shared by people in the group to other people in the community.

**Contacts**

'If you have any questions or concerns regarding the study and would like to talk to someone other than the researcher(s), you are encouraged to contact the Research Subjects' Advocate Line, D-528 Mayo, 420 Delaware Street S.E., Minneapolis, Minnesota, 55455; telephone (612) 625-1650.’

OR

JCRC IRB offices Plot 101 Lubowa off Entebbe Road. Telephone: +256414201148
